# Reversible S-palmitoylation of C4 protein encoded by TYLCCxV orchestrates geminiviral pathogenesis

**DOI:** 10.1007/s44154-026-00308-2

**Published:** 2026-05-06

**Authors:** Yan Xie, Min Zhao, Xianan Liu, Junjie Yan, Wanyi Yang, Yiya Chen, Ming Yang, Xiaowei Wang, Shuai Fu, Xueping Zhou

**Affiliations:** 1 State Key Laboratory of Rice Biology and Breeding, Ministry of Agriculture and Rural Affairs Key Laboratory of Molecular Biology of Crop Pathogens and Insect Pests, Zhejiang Key Laboratory of Biology and Ecological Regulation of Crop Pathogens and Insects, Institute of Biotechnology, Zhejiang University, Hangzhou, 310058 China; 2 State Key Laboratory of Rice Biology and Breeding, Institute of Insect Sciences, Zhejiang University, Hangzhou, 310058 China; 3 State Key Laboratory for Biology of Plant Diseases and Insect Pests, Institute of Plant Protection, Chinese Academy of Agricultural Sciences, Beijing, 100193 China

**Keywords:** S-palmitoylation, Palmitoyl acyltransferases, Depalmitoylases, Geminivirus, Pathogenicity, Plant-virus interaction

## Abstract

Cysteine palmitoylation (S-palmitoylation or S-acylation) is a reversible post-translational modification dynamically controlled by opposing enzymes: palmitoyl acyltransferases (PATs) and depalmitoylases. Despite its established roles in other systems, the mechanistic details of S-acylation in plants, particularly its spatiotemporal regulation during plant-virus interactions, remain poorly understood, largely due to the lack of validated enzyme–substrate pairs. Using the geminivirus tomato yellow leaf curl Chuxiong virus (TYLCCxV) as a model, we show that the viral effector C4 undergoes S-palmitoylation at Cys-4, a modification essential for its plasma membrane anchorage and subsequent mediation of viral pathogenesis. NbPAT4, a palmitoyl acyltransferase from *Nicotiana benthamiana*, catalyzes C4 S-palmitoylation, promoting its membrane localization, protein stability, and viral infection. Conversely, the C4S substitution (C4^C4S^) abolishes S-palmitoylation, leading to cytoplasmic redistribution, protein destabilization, and impaired viral pathogenicity. We further identify NbABHD6 as a depalmitoylase that interacts with C4 and catalyzes its S-depalmitoylation, triggering C4 degradation via the 26S proteasome pathway. This study reveals a regulatory axis in plant-geminivirus interactions, identifying NbPAT4 and NbABHD6 as antagonistic enzymes that dynamically regulate the stoichiometry of C4 S-acylation. These findings support a host–pathogen enzymatic tug-of-war model in which competitive S-palmitoylation homeostasis governs viral subcellular trafficking and pathogenicity.

**Supplementary Information:**

The online version contains supplementary material available at 10.1007/s44154-026-00308-2.

## Introduction

Palmitoylation, a key form of protein lipidation in eukaryotic cells, includes N-palmitoylation, O-palmitoylation, and S-palmitoylation (Yang et al. 2008). Among these, cysteine palmitoylation—also known as S-palmitoylation or S-acylation—involves the covalent attachment of a saturated 16-carbon palmitate to a cysteine (Cys) residue via a thioester bond. This modification is dynamic and reversible due to the intrinsic instability of the thioester linkage (Zaballa and van der Goot 2018). S-acylation plays a central role in regulating membrane association, subcellular trafficking, protein stability, and enzymatic activity under diverse cellular conditions (Linder and Deschenes 2007).

S-acylation is a reversible process that is controlled by two antagonistic enzyme families: palmitoyl acyltransferases (PATs) and depalmitoylases (Magee and Seabra 2005). PATs are characterized by a conserved cysteine-rich domain containing the catalytic DHHC (Asp-His-His-Cys) motif, which mediates the transfer of palmitoyl groups to target proteins (Mitchell et al. 2006). In yeast and mammalian systems, S-acylation has been extensively characterized. In humans, 23 PATs have been identified, many of which are implicated in various diseases, highlighting the critical role of DHHC-PATs in both physiological and pathological contexts (De and Sadhukhan 2018). In contrast, studies of S-acylation in plants remain limited (Hemsley 2020). Nevertheless, bioinformatic analyses have revealed a range of DHHC protein homologs across multiple plant species (Yuan et al. 2013). In *Arabidopsis*, researchers have identified 24 PATs with distinct subcellular localizations (Batistic 2012), providing a foundation for further exploration of S-acylation in plant biology. An increasing number of S-palmitoylated proteins have been identified in rice, maize, and soybean (Yuan et al. 2013; Li et al. 2016). Notably, rice stripe virus (RSV) and potato mop top virus (PMTV) have evolved to exploit host S-acylated proteins to facilitate infection. In *Nicotiana benthamiana*, the RSV-encoded NSvc4 protein interacts with Remorin 1 (NbREM1), disrupts its S-palmitoylation, and promotes its degradation through the autophagy pathway, thereby enhancing RSV infection (Fu et al. 2018). Similarly, PMTV employs its triple gene block 1 (TGB1) protein to interfere with the S-acylation of NbHIPP26 (heavy metal-associated isoprenylated plant protein 26). This disruption enables both NbHIPP26 and TGB1 to translocate to the nucleus via microtubules, ultimately promoting the long-distance movement of PMTV (Cowan et al. 2018).

To date, three plant viral proteins have been reported to undergo S-acylation. AC4 from mungbean yellow mosaic virus (MYMV), which localizes to the plasma membrane via S-acylation, interacts with the RNA interference (RNAi) regulator BARELY ANY MERISTEM 1 (BAM1) and suppresses gene silencing (Carluccio et al. 2018). The S-acylated C4 protein of beet severe curly top virus (BSCTV) interacts with the receptor kinase CLAVATA 1 (CLV1), which regulates meristem maintenance, leading to developmental abnormalities (Li et al. 2018). In barley stripe mosaic virus (BSMV), the S-palmitoylated γb protein recruits TGB1 to chloroplasts and facilitates the switch between viral replication and movement. This process is regulated by the host palmitoyl acyltransferases NbPAT15 and NbPAT21 (Yue et al. 2022).

Depalmitoylases, also known as de-S-acylation enzymes, catalyze the hydrolysis of thioester bonds to remove acyl groups from protein substrates. In mammalian cells, three major classes have been identified: palmitoyl-protein thioesterase 1 (PPT1), the acyl-protein thioesterases APT1 and APT2, and the alpha/beta hydrolase domain-containing protein 17 (ABHD17) family (Won et al. 2018). In contrast, the study of de-S-acylation in plants remains limited. For instance, a putative thioesterase from *Zea mays*, ZmB6T1C9, shares structural homology with mammalian APTs, but its depalmitoylase activity has not been confirmed (Burger et al. 2017). In *Arabidopsis thaliana*, 11 proteins structurally similar to mammalian ABHD17 have been proposed as candidate depalmitoylases, which modulate S-acylation levels and affect the subcellular localization of five immunity-related proteins (Liu et al. 2021). Furthermore, in *Medicago truncatula*, the S-acylated transcription factor MtNAC80 translocates from membranes to the nucleus in response to cold stress via MtAPT1-mediated de-S-acylation (Ye et al. 2024). More recently, ABAPT3 in *Arabidopsis* was identified as the de-S-acylation enzyme targeting the C4 protein encoded by BSCTV (Zhao et al. 2024). To date, this represents the only known case of a plant-virus interaction involving a defined substrate and its specific de-S-acylation enzyme. Despite these advances, the molecular functions and regulatory mechanisms of de-S-acylation in plant remain largely unexplored.

Geminiviruses are a family of plant viruses characterized by single-stranded circular DNA (ssDNA) and are responsible for causing severe diseases in important crops (Varma and Malathi 2003). The largest genus, *Begomovirus*, contains 445 species and is further classified into bipartite and monopartite groups. Bipartite begomoviruses have two circular genomic components, DNA-A and DNA-B, while monopartite begomoviruses possess a single circular genome, similar in size to DNA-A, approximately 2.8 kb (Rojas et al. 2005).

In the ongoing arms race between geminiviruses and plants, geminiviruses have evolved sophisticated strategies to suppress host immunity, including the use of multifunctional effector proteins such as C4. The C4 protein acts as a key virulence factor by serving as a symptom determinant, suppressing transcriptional gene silencing (TGS) and post-transcriptional gene silencing (PTGS), and disrupting multiple layers of plant defense. C4 from BSCTV upregulates RKP, leading to reduced levels of the cell-cycle inhibitors ICK/KRP, which disrupts the mitotic cycle and causes abnormal cell division (Lai et al. 2009). Tomato yellow leaf curl virus (TYLCV) C4 interacts with the receptor kinase BAM1 to block RNAi movement and inhibit PTGS (Rosas-Diaz et al. 2018). Cotton leaf curl Multan virus (CLCMuV) C4 binds to S-adenosyl methionine synthetase (SAMS), a central enzyme in the methylation cycle, thereby inhibiting SAMS activity and suppressing TGS (Ismayil et al. 2018). TYLCV C4 also impairs salicylic acid biosynthesis by hijacking the calcium-sensing receptor CAS, weakening plant immunity (Medina-Puche et al. 2020). Additionally, C4 from tobacco leaf curl Yunnan virus (TbLCYnV) promotes degradation of the sucrose nonfermenting-1-related kinase 1 subunit β (NbSnRK1β2) through direct interaction, thus antagonizing host antiviral responses (Li et al. 2024).

TYLCCxV, a typical monopartite begomovirus recently identified in China, induces severe disease symptoms in plants, including upward leaf curling, crumpling, chlorosis, and stunting. The C4 protein of TYLCCxV acts as a symptom determinant and can trigger virus-like symptoms when expressed via a potato virus X (PVX) vector (Xie et al. 2024). However, the molecular mechanism by which C4 manipulates host factors to regulate plant defense responses remains unclear. Although previous studies have shown that BSCTV C4 and MYMV AC4 undergo S-acylation (Carluccio et al. 2018; Li et al. 2018), their enzymatic regulation and the biological role of the S-acylation cycle in plant-virus interactions remain largely unexplored. In this study, we demonstrate that TYLCCxV C4 undergoes reversible S-acylation at cysteine residue 4. The palmitoyl acyltransferase NbPAT4 catalyzes C4 palmitoylation, promoting its accumulation and localization to the plasma membrane, which enhances viral infection. In contrast, the depalmitoylase NbABHD6 mediates C4 de-S-acylation, leading to its degradation via the 26S proteasome pathway. This dynamic palmitoylation–depalmitoylation cycle of C4 reveals a previously unrecognized regulatory layer in the arms race between geminiviruses and host defenses.

## Results

### S-acylation at cysteine-4 of TYLCCxV C4 protein facilitates its plasma membrane localization and protein accumulation

**Fig. 1:**
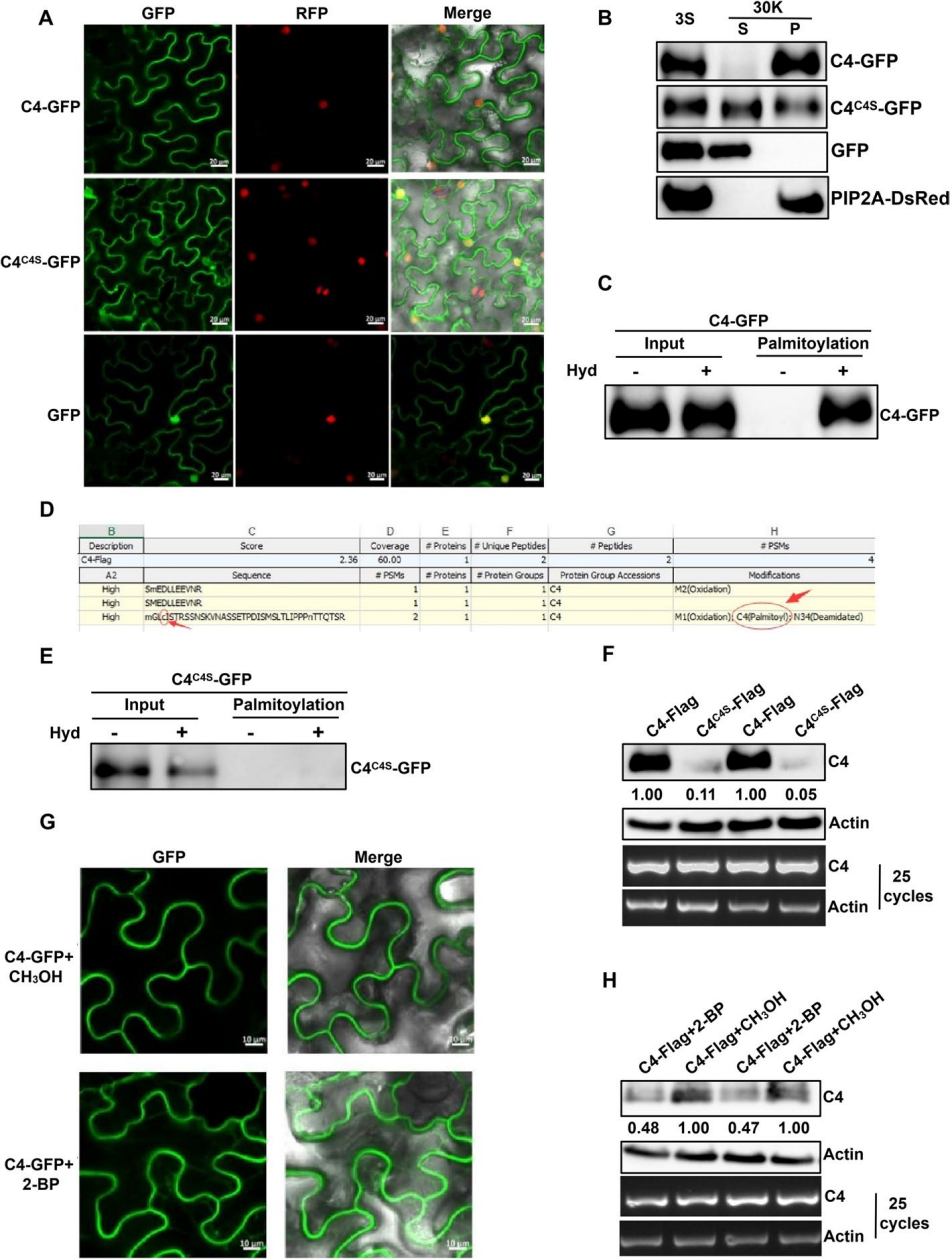
S-acylation at Cysteine-4 in C4 encoded by TYLCCxV promotes plasma membrane localization and protein expression. **A** Representative confocal microscopy images of subcellular localization of C4 and mutant C4^C4S^ in *N. benthamiana* leaf epidermal cells. Transgenic *H2B-RFP N*. *benthamiana* plants were infiltrated with C4-GFP or C4^C4S^-GFP or GFP, respectively. H2B was used as a nucleus marker. Columns from left to right: GFP, RFP, and a GFP/RFP merged field (Merge). Scale bars, 20 μm. **B** Subcellular fractionation analysis of C4 and mutant C4^C4S^. *N. benthamiana* leaves were co-infiltrated with PIP2A-DsRed and C4-GFP, C4^C4S^-GFP, or GFP, respectively. Leave tissues were fractionated into soluble (S) and membrane-enriched (P) fractions after centrifugation at 3,000 g or 30,000 g (30 K). Immunoblot was used to detect anti-GFP or anti-RFP antibodies. GFP and PIP2A-DsRed acted as markers of the soluble and membrane-enriched fractions, respectively. **C** and **E** S-acylation of C4 (**C**) or mutant C4^C4S^ (**E**) proteins detected by the biotin-switch assay. *N. benthamiana* leaves were infiltrated with C4-GFP or C4^C4S^-GFP, and protein extracts were loaded onto NeutraAvidin beads. Immunoblots were conducted with an anti-GFP antibody. Lanes labeled “Palmitoylation” show levels of C4-GFP or C4^C4S^-GFP recovered from Neutravidin beads and indicate Palmitoylation. Hyd, hydroxylamine. **D** Detection of the S-acylation site in C4 via Q-exactive liquid chromatography-tandem mass spectrometry (LC–MS/MS) analysis. *N. benthamiana* leaves were expressed with C4-Flag and protein extracts were analyzed by SDS-PAGE. The red arrow and circle point to the S-acylation site at cysteine-4. **F** Western blot analysis and semi-qRT-PCR assay of C4 and C4^C4S^ protein accumulation and mRNA levels. C4-Flag or C4^C4S^-Flag was infiltrated into opposite halves of the *N. benthamiana* leaves, and protein extracts were detected via Western blots with an anti-Flag antibody. The amount of C4 expression was calculated using Image J 1.8.0, and the data of C4-Flag was set to 1.00. Expression of actin was used as a loading control. Transcription levels of C4 and C4^C4S^ were identified by semi-qRT-PCR. *Actin* was used as the reference gene. **G** and **H** Effects of 2-bromopalmitate (2-BP) on the subcellular localization (**G**) and protein accumulation on the transcription level of C4 (**H**). *N. benthamiana* leaves transiently expressing C4-GFP or C4-Flag were co-infiltrated with 500 µM 2-BP or CH_3_OH (a solution as control). Scale bars, 10 μm (**G**). Samples were detected by Western blot with an anti-Flag antibody. Protein accumulation of C4 was calculated by Image J 1.8.0, and data of C4 with CH_3_OH treatment was set to 1.00. Semi-qRT-PCR detected the transcription level of C4 mRNA (**H**)

TYLCCxV C4 functions as a symptom determinant (Xie et al. 2024), but its underlying mechanism remains poorly understood. To investigate the subcellular localization of C4, we infiltrated leaves of transgenic *N. benthamiana* expressing H2B-RFP with constructs expressing C4-GFP (GFP fused to the C-terminus of C4) or GFP alone. At 48 h post-infiltration (hpi), we used confocal microscopy to visualize GFP and RFP fluorescence in locally infected leaves. C4-GFP fluorescence localized predominantly to the cytoplasm and plasma membrane, with weak nuclear signals, whereas GFP alone was distributed throughout the cytoplasm and nucleus (Fig. 1A). To validate C4 localization, we performed a subcellular fractionation assay. We co-infiltrated *N. benthamiana* leaves with PIP2A-DsRed (a plasma membrane marker) and either C4-GFP or GFP. We then separated total protein extracts into pellet (P30) and supernatant (S30) fractions by centrifugation at 30,000 × g. Western blot analysis showed that C4-GFP co-fractionated with PIP2A-DsRed in the P30 pellet, indicating membrane association, whereas GFP alone appeared exclusively in the S30 supernatant, consistent with its cytosolic localization (Fig. 1B). Together, these results indicate that TYLCCxV C4 predominantly localizes to the plasma membrane.

To investigate the reason for the plasma membrane localization of TYLCCxV C4, we analyzed the C4 protein structure using the online protein transmembrane domain prediction tool (http://www.cbs.dtu.dk/services/TMHMM/). The analysis revealed that C4 does not contain a transmembrane domain. However, two lipid modifications were predicted: N-myristoylation at glycine 2 and S-acylation at Cys-4, as determined by the CSS-Palm 4.0 online server http://csspalm.biocuckoo.org/online.php) (Supplemental Fig. S1). To verify the S-acylation of C4, we infiltrated *N. benthamiana* leaves with C4-GFP and extracted protein samples at 2 days post-infiltration (dpi). We then performed a biotin-switch assay (Hemsley et al. 2008), where the presence of a clear signal in hydroxylamine-treated samples indicates protein S-acylation. A strong S-acylation signal was observed in the hydroxylamine-treated samples, while no signal was detected in the untreated controls. Moreover, the expression levels of C4 were similar in both hydroxylamine-treated and untreated input samples (Fig. 1C), confirming that TYLCCxV C4 undergoes S-acylation. To pinpoint the specific S-acylation site in C4, we performed immunoprecipitation of C4-Flag protein from *N. benthamiana* leaves followed by analysis using Q-Exactive liquid chromatography-tandem mass spectrometry (LC–MS/MS). The results revealed that TYLCCxV C4 is modified by S-acylation at Cys-4 (Fig. 1D). Additionally, we substituted Cys-4 in C4 with serine (C4^C4S^) and analyzed the S-acylation levels of the mutant C4 using the biotin-switch assay. No S-acylation signal was detected in the C4^C4S^ mutant following hydroxylamine treatment (Fig. 1E). This result indicates that the mutation of Cys-4 to serine completely abolishes S-acylation, further confirming that C4 undergoes S-acylation at Cys-4.

To assess the impact of S-acylation on the subcellular localization of TYLCCxV C4, we infiltrated transgenic *N. benthamiana* leaves expressing H2B-RFP with either C4-GFP or C4^C4S^-GFP (GFP fused to the C-terminus of C4^C4S^). The fluorescence of C4-GFP was predominantly detected at the plasma membrane, while C4^C4S^-GFP fluorescence was observed in the cytoplasm and nucleus, similar to GFP alone (Fig. 1A). We further confirmed these observations through subcellular fractionation. C4^C4S^-GFP appeared in both the pellet (P30) and supernatant (S30) fractions (Fig. 1B), reinforcing the conclusion that S-acylation is crucial for the plasma membrane anchoring of C4. Next, we investigated whether S-acylation influenced C4 turnover. C4-Flag and C4^C4S^-Flag (Flag fused to the C-terminus of C4 or C4^C4S^) were agroinfiltrated into opposite halves of the same *N. benthamiana* leaf. Protein expression levels were quantified using ImageJ 1.8.0, with C4-Flag expression set to 1.00. Western blot analysis revealed a significant reduction in C4^C4S^-Flag protein levels (0.11; 0.05) compared to the wild-type C4-Flag (1.00; 1.00) in the inoculated leaves (Fig. 1F). To determine whether the decrease in C4 protein levels was due to changes in transcript abundance, we extracted total RNA from the same plant samples. Semi-quantitative RT-PCR analysis showed that the transcript levels of both wild-type C4 and C4^C4S^ were comparable (Fig. 1F). Together, these results demonstrate that TYLCCxV C4 localizes to the plasma membrane via S-acylation at Cys-4, and that S-acylation enhances C4 protein accumulation and stability.

To investigate the impact of S-acylation inhibition on the subcellular localization and stability of C4 protein, we treated *N. benthamiana* plants expressing C4-GFP or C4-Flag with 500 µM 2-bromopalmitate (2-BP), a specific inhibitor of protein palmitoylation (Hemsley et al. 2005). Plants co-infiltrated with C4-GFP or C4-Flag and methanol (CH_3_OH), the solvent for 2-BP, served as negative controls. Under 2-BP treatment, the C4-GFP signal was detected in the cytoplasm, whereas in the control (CH_3_OH-treated) plants, the C4-GFP signal was primarily localized at the plasma membrane (Fig. 1G). Western blot analysis revealed that C4 protein accumulation was significantly reduced in the 2-BP-treated plants (0.48; 0.47) compared to the control group (1.00; 1.00). This decrease in protein accumulation was not due to reduced transcription, as semi-quantitative RT-PCR showed comparable mRNA levels between the two treatments (Fig. 1H). These results suggest that the palmitoylation inhibitor 2-BP affects C4 protein localization and accumulation by reducing its S-acylation level. Together, these experiments provide compelling evidence that TYLCCxV C4 undergoes S-acylation at Cys-4 in vivo, and that S-acylation is essential for C4's plasma membrane localization and protein stability.

### S-acylation of C4 protein affects TYLCCxV pathogenicity

**Fig. 2:**
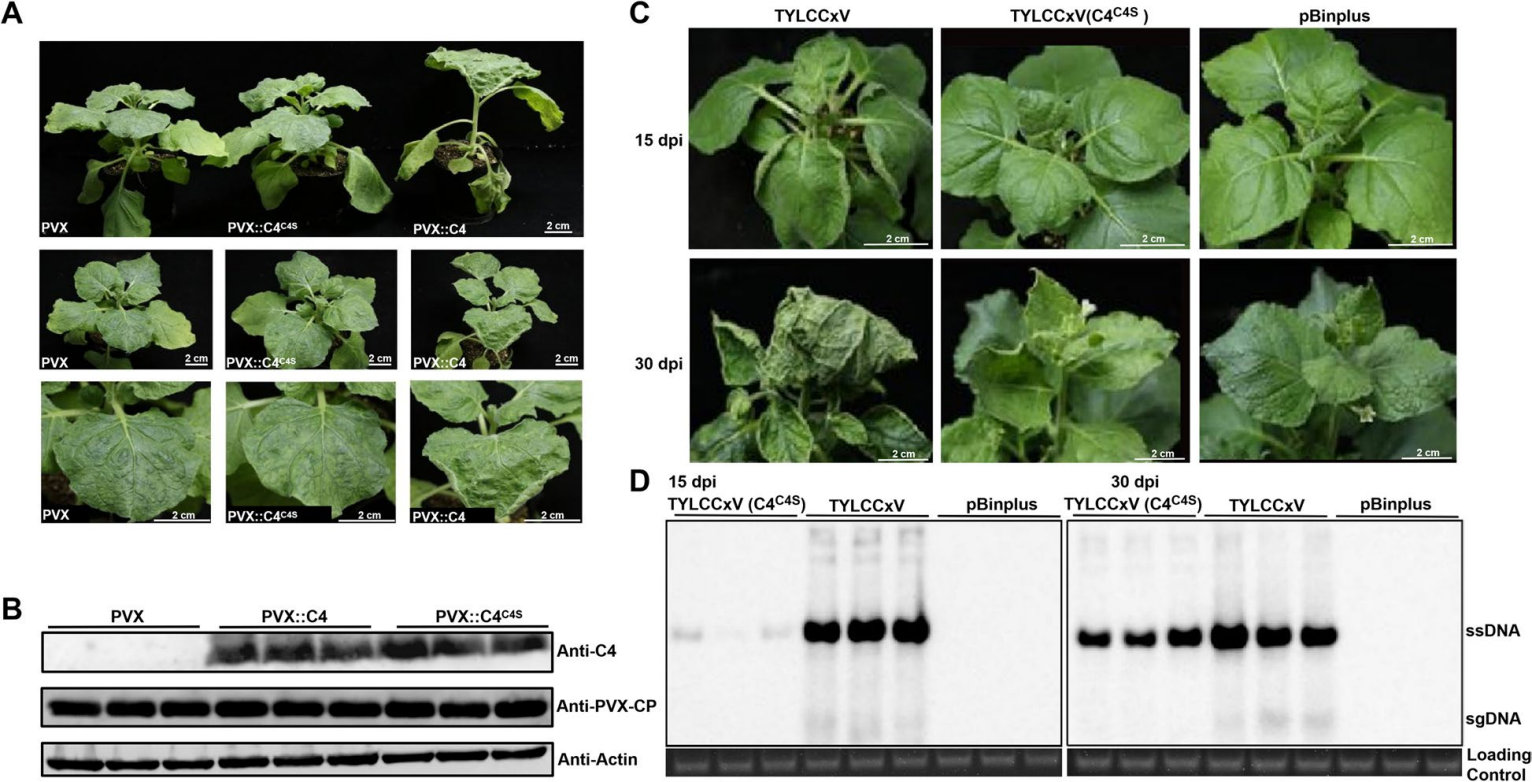
S-acylation of the C4 protein regulates viral infection. **A** Symptoms of *N. benthamiana* plants inoculated with PVX, PVX::C4^C4S^, and PVX::C4 at 14 dpi. Scale bars, 2 cm. **B** Western blot analysis of C4 expression levels and PVX accumulation in agroinfiltrated leaf patches shown in panel A. Samples were analyzed with immunoblots using anti-C4 or anti-PVX-CP. Actin detected with an anti-Actin antibody was used as a loading control. **C** Symptoms of *N. benthamiana* plants infected with TYLCCxV or mutant TYLCCxV (C4^C4S^) at 15/30 dpi. Scale bars, 2 cm. **D** Southern blot analysis of TYLCCxV DNA accumulation in *N. benthamiana* plants shown in panel C at 15/30 dpi. Total genomic DNA (10 μg per lane) from a mixture of three seedlings was used in the Southern blot. The blot was hybridized with a TYLCCxV coat protein (CP) probe. Ethidium-bromide-stained gel shown below the blots provide a DNA-loading control. Positions of single-stranded (ss) DNA and subgenomic (sg) DNA forms are indicated

To investigate the role of S-acylation of C4 in viral infection, *Agrobacterium tumefaciens* harboring C4 or its mutant C4^C4S^ was used to inoculate *N. benthamiana* plants via a PVX-based vector (pGR106). Plants inoculated with PVX::C4 developed systemic upward leaf curling and stem elongation by 14 dpi, while only mild chlorotic symptoms, similar to the negative control PVX, were observed in PVX::C4^C4S^-infected plants (Fig. 2A). Western blot analysis of protein extracts from these plants using anti-C4 and anti-PVX-CP antibodies revealed that the expression levels of C4 were comparable between the PVX::C4- and PVX::C4^C4S^-inoculated plants. Furthermore, the accumulation of PVX CP was also similar across all plants inoculated with PVX-based constructs (Fig. 2B).

To further explore the biological function of C4 S-acylation during viral infection, an infectious clone of the TYLCCxV mutant (C4^C4S^) was generated, in which serine replaced cysteine at the fourth position of the C4 protein. *N. benthamiana* plants were inoculated with the TYLCCxV infectious clone, TYLCCxV (C4^C4S^), or an empty pBinPLUS vector. Notably, plants inoculated with TYLCCxV (C4^C4S^) exhibited milder symptoms than those infected with the wild-type TYLCCxV. Systemic leaves of TYLCCxV (C4^C4S^)-inoculated plants displayed no obvious viral symptoms, resembling the symptoms in pBinPLUS-inoculated plants. In contrast, TYLCCxV-infected plants developed upward leaf curling by 15 dpi. At 30 dpi, TYLCCxV-infected plants showed severe upward leaf curling and vein swelling, while TYLCCxV (C4^C4S^)-infected plants exhibited only slight upward curling and stunted growth (Fig. 2C). Southern blot analysis revealed that viral signals were weakly detectable in TYLCCxV (C4^C4S^)-infected plants at 15 dpi, with further reductions observed at 30 dpi compared to TYLCCxV-infected plants. No viral signal was detected in pBinPLUS-inoculated plants (Fig. 2D). These results suggest that S-acylation of C4 at Cys-4 plays a critical role in regulating TYLCCxV infection.

### TYLCCxV C4 interacts with palmitoyl acyltransferase (NbPAT4) *in vitro* and *in vivo*

**Fig. 3:**
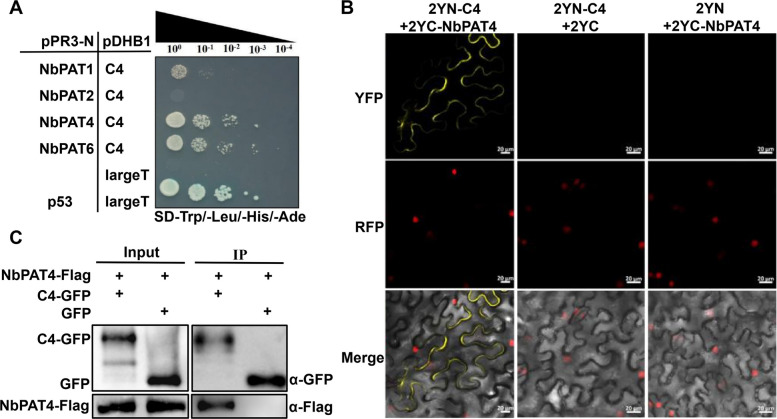
Interaction of C4 with NbPAT4 in vitro and in vivo. **A** Yeast Split-ubiquitin assay of the interaction between TYLCCxV C4 and four palmitoyl acyltransferases (NbPAT1-2, NbPAT4, and NbPAT6). The yeast strain NMY51 co-transformed with the indicated plasmids was subjected to tenfold serial dilutions and grown on a SD/-Trp/-Leu/-His/-Ade medium. Strains carried combinations of pPR3-N-p53/pDHB1-largeT were used as positive control, and those with empty pPR3-N/PDHB1-largeT were used as negative control. **B** BiFC analysis showing the interaction of C4 and NbPAT4. The transgenic *H2B-RFP N*. *benthamiana* plants were co-infiltrated with 2YN-C4 and 2YC-NbPAT4, 2YN-C4, and 2YC, or 2YN and 2YC-NbPAT4, respectively. H2B was used as a nucleus marker. Columns from top to bottom: YFP, RFP, and Merge. Scale bars, 20 μm. **C** Interaction between TYLCCxV C4 and NbPAT4 via the Co-IP assay. *N. benthamiana* leaves co-infiltrated the NbPAT4-Flag with C4-GFP or GFP. Total protein extracts were incubated with anti-GFP mAb-magnetic beads. The input and the co-immunoprecipitated proteins were detected by Western blot with an anti-GFP or anti-Flag antibody

To investigate whether TYLCCxV C4 interacts with palmitoyl acyltransferases in *N. benthamiana*, we screened four palmitoyl acyltransferases (NbPAT1, NbPAT2, NbPAT4, NbPAT6) using a Yeast Split-ubiquitin assay. The candidate enzymes were cloned into the pPR3-N vector, and TYLCCxV C4 was introduced into the pDHB1 vector. Both constructs were co-transformed into the Yeast strain NMY51. Positive and negative controls were established using the pPR3-N-p53/pDHB1-largeT combination and the empty pPR3-N/pDHB1-largeT, respectively. The results showed that yeast strains harboring pPR3-N-NbPAT4 or pPR3-N-NbPAT6 and pDHB1-C4 grew on the SD/-Trp/-Leu/-His/-Ade medium, similar to the positive control (Fig. 3A). Given their functional redundancy, we selected NbPAT4 for further analysis using a Bimolecular Fluorescence Complementation (BiFC) assay in transgenic *H2B-RFP N. benthamiana* leaves. TYLCCxV C4 and NbPAT4 were fused to the split N- or C-terminal fragments of the yellow fluorescent protein (YFP) to generate 2YN-C4 and 2YC-NbPAT4 constructs, respectively. Confocal microscopy revealed YFP fluorescence at the cytomembrane but minimal fluorescence in the nucleus (Fig. 3B), indicating that TYLCCxV C4 interacts with NbPAT4 in vivo. To further confirm the interaction, a co-immunoprecipitation (Co-IP) assay was performed in *N. benthamiana* leaves co-infiltrated with NbPAT4-Flag and C4-GFP or GFP. Protein extracts were immunoprecipitated using GFP mAb-magnetic beads, followed by Western blot analysis with anti-GFP or anti-Flag antibodies. The Co-IP assay showed a specific interaction between NbPAT4-Flag and C4-GFP but not GFP (Fig. 3C). Together, these results demonstrate that TYLCCxV C4 directly interacts with NbPAT4 both in vitro and in vivo.

### NbPAT4 contributes to TYLCCxV infection and is involved in the S-acylation of C4

**Fig. 4:**
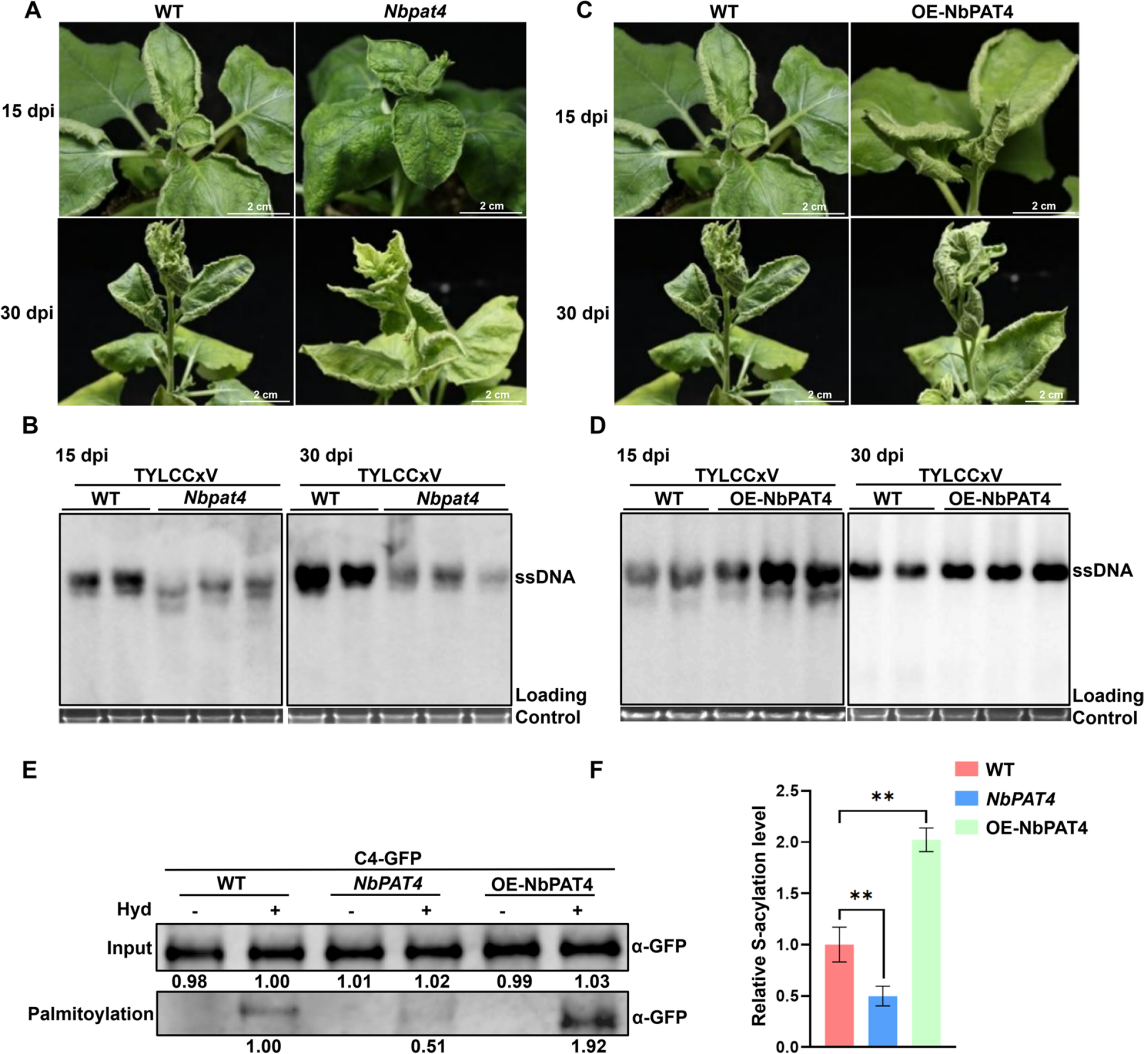
NbPAT4 modulates TYLCCxV infection and participates in S-palmitoylation of C4. **A** and** C** Symptoms induced by TYLCCxV in wild type (WT) and transgenic *Nbpat4* (**A**) or OE-NbPAT4 *N. benthamiana* (**C**) at 15/30 dpi. Scale bars, 2 cm. **B** and **D** Southern blot analysis of TYLCCxV DNA accumulation in *N. benthamiana* plants shown in panel a (**B**) or panel c (**D**) at 15/30 dpi. Total genomic DNA (10 μg per lane) from a mixture of three seedlings was used in Southern blot. The blot was probed with the CP of TYLCCxV. The position of the ssDNA form is indicated. **E** S-acylation levels of C4 protein detected by the biotin-switch assay in wild type *N. benthamiana* (WT), the transgenic *Nbpat4*, or OE-NbPAT4 plants. Samples were loaded onto NeutraAvidin beads and detected via an immunoblot with the anti-GFP antibody. Lanes labeled “Palmitoylation” show levels of C4-GFP recovered from NeutrAvidin beads and indicate Palmitoylation. Hyd, hydroxylamine. The S-acylation level and protein accumulation of C4 are calculated using Image J 1.8.0, and the data of C4 with Hyd treatment in wild type plants was set to 1.00. **F** S-acylation levels of C4 shown in panel e quantified using GraphPad Prism 8. The S-acylation level of C4 in wild type *N. benthamiana* plants was set to 1.0. Data are presented as means and SD of three biological replicates. Two asterisks mark significant differences between two treatments at *p* < 0.01via Student’s *t*-test

To investigate the biological role of NbPAT4 in viral infection, we infected transgenic *Nicotiana benthamiana* plants with TYLCCxV. These plants included *NbPAT4* knockout (*Nbpat4*) and overexpressing (OE-NbPAT4) lines. At 15 dpi, the *Nbpat4* plants exhibited mild, slight up-curling of leaves, while wild-type (WT) plants displayed severe, upward curling. By 30 dpi, WT plants showed pronounced upward curling, while *Nbpat4* plants exhibited less severe symptoms (Fig. 4A). Southern blot analysis revealed that *Nbpat4* plants had lower levels of viral genomic DNA compared to WT plants at both 15 and 30 dpi (Fig. 4B). In contrast, OE-NbPAT4 plants showed more severe symptoms, including greater upward leaf curling, vein swelling, and leaf clustering, at both 15 and 30 dpi, compared to TYLCCxV-inoculated WT plants (Fig. 4C). Additionally, Southern blot analysis demonstrated higher viral genomic DNA levels in OE-NbPAT4 plants than in WT plants (Fig. 4D). Collectively, these findings suggest that NbPAT4 enhances TYLCCxV infection in *N. benthamiana*.

To further explore whether NbPAT4 contributes to the S-acylation of the TYLCCxV C4 protein, we measured the S-acylation levels of C4 protein in C4-GFP-infiltrated WT, *Nbpat4*, and OE-NbPAT4 plants using a biotin-switch assay. As shown in Fig. 4E, C4-GFP was detected at similar levels in the input samples from WT, *Nbpat4*, and OE-NbPAT4 plants, regardless of whether they were treated with Hyd. Importantly, S-acylation was only observed following Hyd treatment, confirming that C4 undergoes S-acylation. S-acylation levels of C4 protein in *Nbpat4* plants were approximately 0.51 (compared to WT plants at 1.00), while in OE-NbPAT4 plants, levels increased to 1.92. These results were confirmed using three independent biological replicates and statistically analyzed with GraphPad Prism 8 (Fig. 4F). Together, these data indicate that NbPAT4, a palmitoyl acyltransferase, is directly involved in the S-acylation of C4.

### TYLCCxV C4 interacts with de-S-acylation enzyme NbABHD6 *in vitro *and *in vivo*

**Fig. 5:**
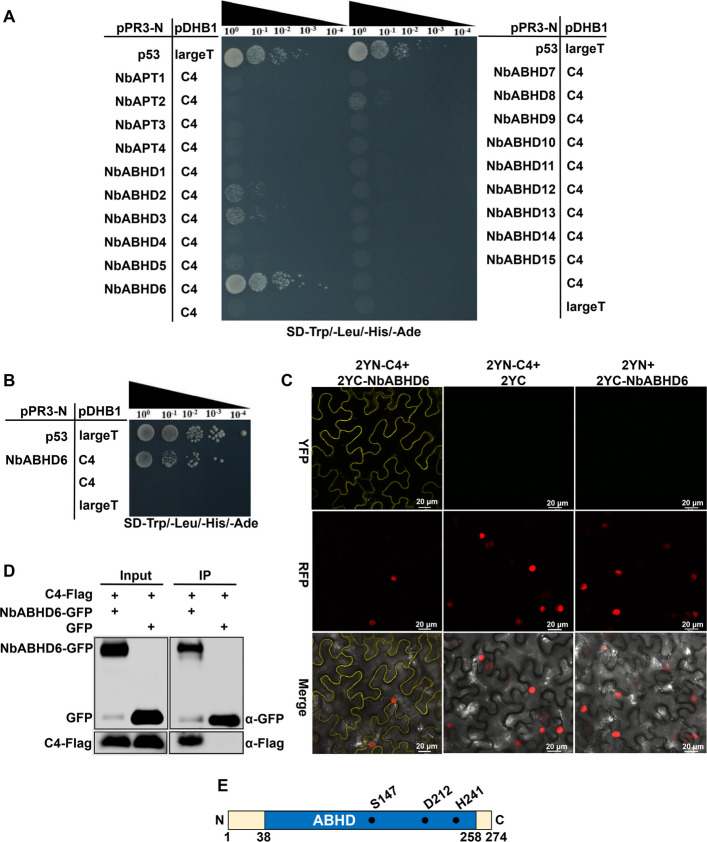
Interaction of C4 with NbABHD6 in vitro and in vivo and a schematic diagram of NbABHD6. **A** Screening of de-S-acylation enzymes interacting with TYLCCxV C4 by the Yeast Split-ubiquitin assay. Strains carried combinations of pPR3-N-p53/pDHB1-largeT and the empty pPR3-N/pDHB1-C4 or largeT were used as positive and negative controls, respectively. **B** Yeast Split-ubiquitin assay of the interaction with TYLCCxV C4 and NbABHD6. **C** Interaction of TYLCCxV C4 and NbABHD6 via the BiFC assay. Transgenic *H2B-RFP N*. *benthamiana* plants were co-infiltrated with 2YN-C4 and 2YC-NbABHD6, 2YN-C4 and 2YC, or 2YN and 2YC-NbABHD6, respectively. Scale bars, 20 μm. **D** Co-IP assay showing the interaction of TYLCCxV C4 and NbABHD6. *N. benthamiana* leaves were co-infiltrated C4-Flag with NbABHD6-GFP or GFP, and protein extracts are incubated with anti-GFP mAb-magnetic beads. The input and co-immunopreciptated proteins are detected by immunoblot with an anti-GFP or anti-Flag antibody. **E** Schematic diagram of the NbABHD6 protein structure. ABHD: αlpha/beta hydrolase domain. Numbers represent the amino acid positions of the NbABHD6 protein. Three residues (S147, D212, and H241) involved in catalysis of NbABHD6 are indicated by the black spots. S147, serine at 147; D212, aspartate at 212; H241, histidine at 241

Protein palmitoylation is a reversible lipid modification involving the processes of palmitoylation and depalmitoylation, which are mediated by palmitoyl acyltransferases and depalmitoylases. To identify the depalmitoylase interacting with TYLCCxV C4, we amplified 19 de**-**S-acylation enzymes, including NbAPT1-4 and NbABHD1-15, from a *N. benthamiana* cDNA library (Supplemental Table S1). These enzymes were then fused to the pPR3-N vector for a Yeast Split-ubiquitin assay. Positive and negative controls consisted of strains carrying pPR3-N-p53/pDHB1-largeT and empty pPR3-N/pDHB1-largeT or C4, respectively. The assay results showed that the strain carrying pPR3-N-NbABHD6/pDHB1-C4 grew robustly, similar to the positive control, whereas other strains failed to grow (Fig. 5A, B).

We further validated this interaction using a BiFC assay. In this experiment, 2YN-C4 and 2YC-NbABHD6 (where NbABHD6 was fused to 2YC) were co-expressed in transgenic *H2B-RFP N. benthamiana*. Confocal fluorescence imaging revealed a strong YFP fluorescence signal in the cytoplasm of cells co-expressing 2YN-C4 and 2YC-NbABHD6, with no signal detected in the negative controls. Notably, the fluorescence was absent in the nucleus (Fig. 5C). Similar results were obtained through Co-IP in vivo. *N. benthamiana* leaves were co-expressed with C4-Flag and NbABHD6-GFP or GFP (as a negative control). Co-IP products were analyzed by Western blot using anti-GFP and anti-Flag antibodies. Both NbABHD6-GFP, GFP, and C4-Flag were detected in the input samples. Importantly, C4-Flag was specifically co-immunoprecipitated by NbABHD6-GFP but not by GFP alone (Fig. 5D). These findings confirm a specific and direct interaction between TYLCCxV C4 and NbABHD6 both in vitro and in vivo.

The mammalian ABHD17 family contains a conserved alpha/beta hydrolase domain (ABHD) with a catalytic triad composed of serine (S), aspartate (D), and histidine (H) residues. This triad is essential for its de-S-acylation activity (Yokoi et al. 2016). Structural analysis, performed using the InterPro website (https://www.ebi.ac.uk/interpro), revealed that the NbABHD6 protein consists of 274 amino acids and contains a typical ABHD region. We aligned the amino acid sequences of ABHDs from rat, *N. benthamiana*, and *Arabidopsis* using Mega 7.0. The alignment showed that these ABHD17 family proteins share highly conserved catalytic triad residues. For NbABHD6, the catalytic triad is composed of S147, D212, and H241 (Fig. 5E, S2), indicating that NbABHD6 may follow a similar reaction mechanism to its homologs.

### NbABHD6 interferes with depalmitoylation of TYLCCxV C4

**Fig. 6:**
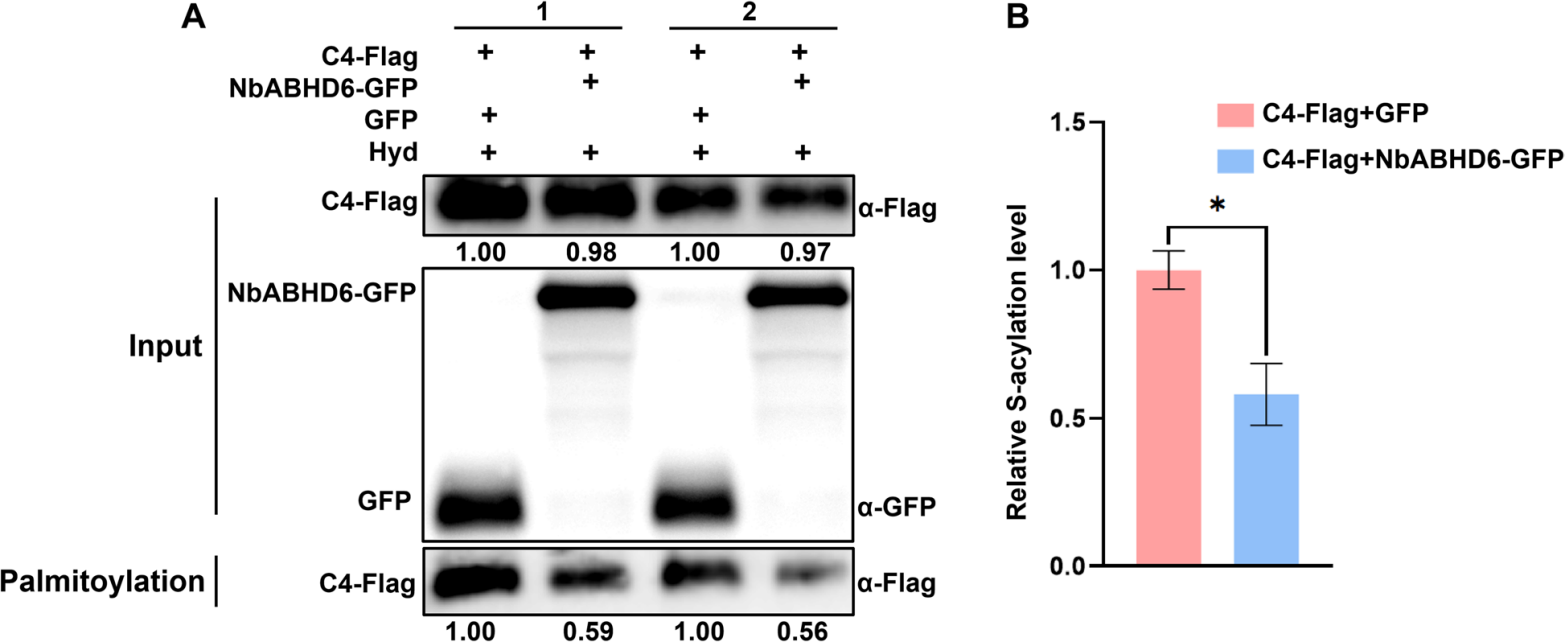
NbABHD6 mediates depalmitoylation of TYLCCxV C4. **A** S-acylation level of the C4 protein as detected by the biotin-switch assay in *N. benthamiana* leaves co-expressing C4-Flag with NbABHD6-GFP or GFP. The S-acylation level of C4 were calculated using Image J 1.8.0, and the data of C4-Flag with the GFP treatment was set to 1.00. Lanes labeled “Palmitoylation” show levels of C4-Flag recovered from the NeutrAvidin beads and indicate Palmitoylation. **B** GraphPad Prism 8 analysis of S-acylation levels of C4 shown in panel A. The data in the C4-Flag and GFP treatments were set to 1.0. Data are presented as means and SD of three biological replicates. Asterisks mark differences between two treatments at *p* < 0.1 via Student’s *t*-test

To assess whether the de-S-acylation enzyme NbABHD6 is involved in the depalmitoylation of C4, we transiently co-expressed C4-Flag with NbABHD6-GFP or GFP (as a control) in the opposite halves of *N. benthamiana* leaves. Following this, we performed a biotin switch assay. The results showed that C4-Flag co-expressed with either NbABHD6-GFP or GFP accumulated at similar levels in the input protein samples (1.00, 0.98, 1.00, 0.97). C4-Flag was detected only following Hyd treatment, indicating that the C4 protein undergoes S-acylation. When comparing the S-acylation levels of C4-Flag co-expressed with NbABHD6-GFP to the control (1.00), the S-acylation levels were significantly reduced to 0.59 or 0.56 (Fig. 6A). GraphPad Prism 8 analysis confirmed these findings, showing that S-acylation of C4 was significantly inhibited when co-expressed with NbABHD6 but not with GFP (Fig. 6B). These results demonstrate that NbABHD6 promotes the de-S-acylation of TYLCCxV C4.

### NbABHD6-mediated depalmitoylation promotes degradation of TYLCCxV C4 protein via the 26S proteasome pathway

**Fig. 7:**
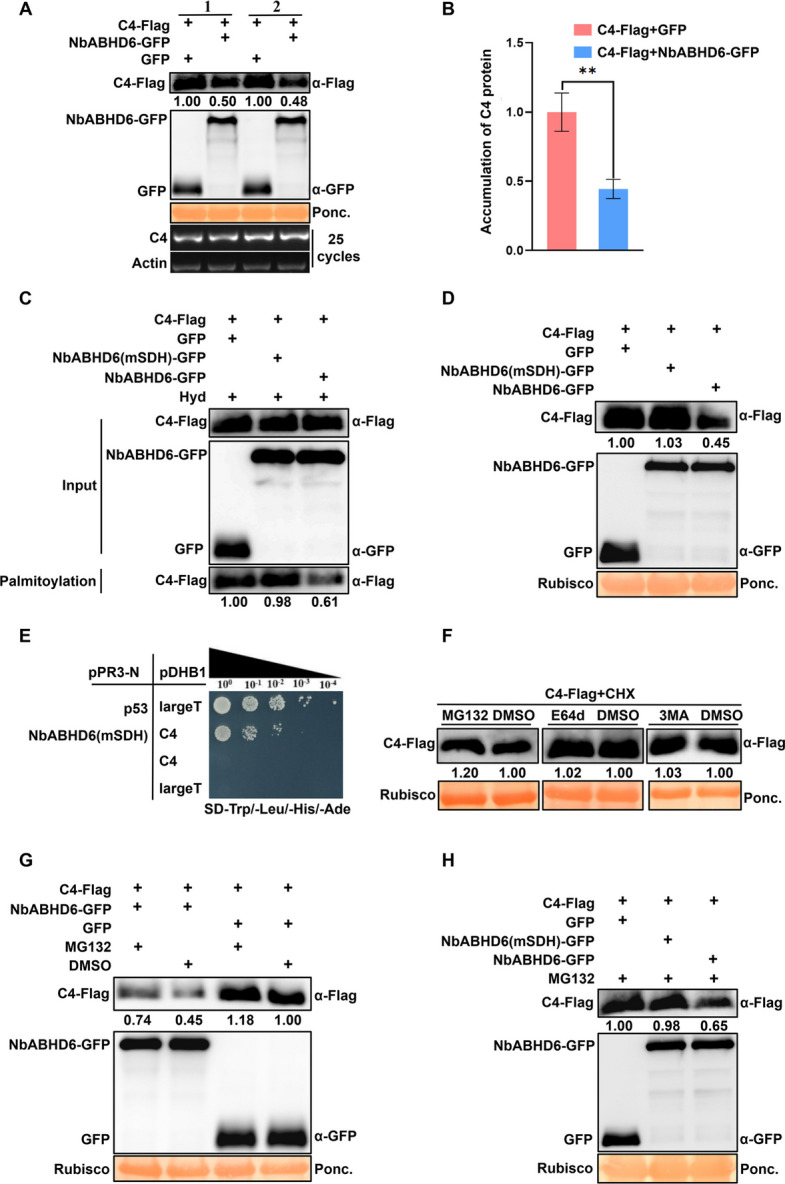
NbABHD6 enhances degradation of TYLCCxV C4 protein by the 26S proteasome pathway. **A** Western blot analysis and semi-qRT-PCR assay of the TYLCCxV C4 protein and mRNA levels in C4-Flag with NbABHD6-GFP or GFP co-infiltrated *N. benthamiana* leaves. Samples were detected via immunoblot with anti-Flag or anti-GFP antibodies. The protein accumulation of C4-Flag was calculated by Image J 1.8.0, and the data of C4 co-infiltrated with GFP were set as 1.00. Ponceau red stained Rubisco was used as a loading control. Transcription levels of C4 were identified by semi-qRT-PCR. *Actin* was used as the reference gene. **B** Analysis of C4 protein accumulation shown in panel a using GraphPad Prism 8. The data in the C4-Flag and GFP treatments were set to 1.0. Data are presented as means and SD of three biological replicates. Two asterisks mark differences between two treatments at *p* < 0.01 via Student’s *t*-test. **C** and **D** S-acylation levels (**C**) and protein accumulation levels (**D**) of the C4 protein detected by the biotin switch assay and Western blot analysis. *N. benthamiana* was co-infiltrated with C4-Flag and GFP, NbABHD6 (mSDH)-GFP, or NbABHD6-GFP, respectively. Protein extracts were analyzed by Western blot with anti-Flag or anti-GFP antibodies. S-acylation and protein accumulation levels of C4 were calculated using Image J 1.8.0, and the data of C4 co-expressed with GFP was set to 1.00. Lanes labeled “Palmitoylation” show the levels of C4-Flag recovered from the NeutrAvidin beads and indicate Palmitoylation. **E** Interaction of NbABHD6 (mSDH) and C4 by the Yeast Split-ubiquitin assay. **F** Semi-in vivo degradation inhibition analysis of C4 using the protease inhibitors MG132, E64d, or 3MA. The *N. benthamiana* leaves transiently expressing C4-Flag were treated with 100 μM CHX together with 100 μM MG132, 100 μM E64d, 5 mM E64d, or DMSO. The relative accumulation level of C4 was calculated using Image J 1.8.0, and the data of C4 in DMSO treatments was set as 1.00. **G** Semi-in vivo C4 protein stability assay by Western blot. *N. benthamiana* leaves transiently co-expressed C4-Flag with NbABHD6-GFP or GFP were treated with 100 μM MG132 or DMSO, respectively. The amount of C4 expressing was calculated using Image J 1.8.0, and the data of C4 co-infiltrated with GFP in DMSO-treated leaves was set as 1.00. **H** Protein accumulation of C4 analyzed by Western blot. *N. benthamiana* co-expressed with C4-Flag and GFP, NbABHD6 (mSDH)-GFP, or NbABHD6-GFP were treated with 100 μM MG132. The accumulation level of C4 was calculated using Image J 1.8.0, and the data of C4 co-infiltrated with GFP was set as 1.00

To determine whether NbABHD6 affects C4 protein accumulation, we co-expressed C4-Flag with NbABHD6-GFP or GFP in the opposite halves of *N. benthamiana* leaves. Protein levels were analyzed at 48 hpi using immunoblotting with anti-Flag or anti-GFP antibodies. As shown in Fig. 7A, C4 protein accumulation in plants co-expressing C4-Flag and NbABHD6-GFP was reduced to 0.50 or 0.48 compared to plants co-expressing C4-Flag and GFP (1.00). Semi-quantitative RT-PCR confirmed that this difference was not due to reduced transcription, as the mRNA levels of C4 were similar across treatments. GraphPad Prism 8 analysis corroborated these findings (Fig. 7B), further validating the role of NbABHD6 in reducing C4 accumulation.

Our bioinformatics analysis suggested that S147, D212, and H241 form the catalytic triad of NbABHD6 (Fig. 5E). To investigate whether these residues contribute to the depalmitoylase activity of NbABHD6, we simultaneously substituted S147, D212, and H241 with alanine, generating a mutant NbABHD6 (mSDH). We then co-infiltrated C4-Flag with GFP, NbABHD6 (mSDH)-GFP, or NbABHD6-GFP into *N. benthamiana*. The biotin switch assay confirmed the S-acylation of C4, as evidenced by the presence of C4-Flag bands following Hyd treatment. The S-acylation level of C4-Flag co-expressed with NbABHD6 (mSDH)-GFP was similar (0.98) to the control group (1.00), while co-expression with NbABHD6-GFP reduced the S-acylation level to 0.61 (Fig. 7C). These results suggest that NbABHD6 (mSDH) lacks the ability to reduce the S-acylation level of C4, in contrast to NbABHD6. Western blot analysis revealed no significant difference in C4-Flag protein accumulation between plants co-expressing C4-Flag with NbABHD6 (mSDH)-GFP (1.03) and GFP (1.00) (Fig. 7D). These findings, together with the biotin switch assay, demonstrate that NbABHD6 (mSDH), with mutations at S147, D212, and H241, lost its de-S-acylation activity and no longer affected the S-acylation level of C4. Consequently, NbABHD6 (mSDH) had no impact on C4 protein accumulation. Our data further support the conclusion that the catalytic triad—composed of S147, D212, and H241—is essential for the depalmitoylation activity of NbABHD6. The effect of NbABHD6 on C4 accumulation is therefore mediated by its depalmitoylase activity. To investigate whether the loss of depalmitoylase activity in NbABHD6 affects its interaction with C4, we fused NbABHD6 (mSDH) to the pPR3-N vector. The Yeast Split-ubiquitin assay showed that NbABHD6 (mSDH) still interacted with C4 (Fig. 7E), suggesting that the depalmitoylase activity of NbABHD6 is not required for its interaction with C4.

Proteins in plants are primarily degraded via the 26S proteasome or autophagy pathways. To identify the degradation pathway of C4 in *N. benthamiana*, we treated plant leaves expressing C4-Flag with inhibitors of these pathways. The 26S proteasome inhibitor MG132, the autophagy inhibitors E64d and 3MA, and DMSO (as a control) were used. Leaves were treated with 100 μM cycloheximide (CHX) and 100 μM MG132, 100 μM E64d, 5 mM 3MA, or DMSO. Western blot analysis revealed that C4 expression was higher in MG132-treated leaves (1.20) compared to DMSO-treated leaves (1.00). In contrast, the levels of C4 remained similar in leaves treated with E64d (1.02), 3MA (1.03), or DMSO (1.00) (Fig. 7F), suggesting that C4 is degraded via the 26S proteasome pathway.

To determine whether NbABHD6 promotes C4 degradation via the 26 proteasome pathway, we co-infiltrated *N. benthamiana* plants with C4-Flag and either NbABHD6-GFP or GFP, followed by treatment with 100 μM MG132 or DMSO. As shown in Fig. 7G, Western blot analysis revealed that, in GFP co-expression controls, the C4-Flag protein level increased in MG132-treated plants (1.18) compared to DMSO-treated plants (1.00), confirming that MG132 inhibits C4 degradation, and thus C4 is degraded via the 26S proteasome pathway. In DMSO-treated leaves, the accumulation of C4-Flag in plants co-expressing NbABHD6-GFP was reduced to 0.45, confirming that NbABHD6 decreases C4 protein levels. In contrast, in MG132-treated leaves, the accumulation of C4-Flag co-expressed with NbABHD6-GFP was 0.74, significantly higher than in the DMSO-treated group (0.45), indicating that NbABHD6 modulates C4 degradation through the 26S proteasome pathway. Notably, C4-Flag accumulation in MG132-treated leaves co-expressing NbABHD6-GFP (0.74) remained lower than in the GFP control group (1.18). This indicates that NbABHD6 reduces C4 accumulation even when proteasome is inhibited, implying the existence of other regulatory mechanisms—a possibility consistent with C4's multifunctional nature. The ratio of C4-Flag accumulation in MG132 versus DMSO-treated plants was 1.64 (0.74/0.45) for NbABHD6-GFP co-expression, compared to 1.18 (1.18/1.00) in the GFP control, further supporting that NbABHD6-mediated depalmitoylation promotes C4 degradation via the 26S proteasome pathway. Additionally, Western blot analysis in Fig. 7H demonstrated that, in the presence of MG132, the accumulation of C4-Flag with NbABHD6 (mSDH)-GFP was similar (0.98) to the control group (1.00), while C4-Flag accumulation was reduced to 0.65 when co-expressed with NbABHD6-GFP. These findings indicate that NbABHD6, but not NbABHD6 (mSDH), promotes C4 degradation via the 26S proteasome pathway, and this effect is mediated by its depalmitoylase activity.

## Discussion

Our previous studies demonstrated that TYLCCxV C4 acts as a symptom determinant, causing upward leaf curling and stem elongation when expressed heterologously in PVX (Xie et al. 2024). However, the precise mechanism by which C4 interacts with host factors remains unclear. In this study, we found that C4 primarily localizes to the plasma membrane through S-acylation at Cys-4 (Supplemental Fig. S1, Fig. 1A–E). Further investigation revealed that S-acylation of C4 regulates both its subcellular localization and protein stability (Fig. 1A, B, F–H), consistent with the well-established role of S-palmitoylation in modulating the membrane association and stability of regulatory proteins (Linder and Deschenes 2007). However, the biological function of C4 S-acylation remains unclear. Our findings showed that PVX::C4^C4S^-infected plants exhibited no viral symptoms, and *N. benthamiana* leaves inoculated with TYLCCxV (C4^C4S^) displayed milder symptoms and lower viral accumulation compared to wild-type TYLCCxV-infected plants (Fig. 2). These results suggest that C4 S-acylation is associated with pathogenicity and raise the question of how this post-translational modification is enzymatically regulated. Next, we identified NbPAT4 as a protein that interacts with C4 (Fig. 3). Functional analyses revealed that NbPAT4 influences TYLCCxV pathogenicity: viral symptoms and accumulation were reduced in NbPAT4 knockdown plants (*Nbpat4*), while overexpression of NbPAT4 (OE-NbPAT4) enhanced viral pathogenicity (Fig. 4A–D). Importantly, reduced S-acylation levels of C4 in *Nbpat4* plants and elevated S-palmitoylation in OE-NbPAT4 plants (Fig. 4E, F) provide evidence that NbPAT4 enzymatically regulates C4 S-acylation. However, direct biochemical validation of PAT4-mediated catalysis (e.g., via in vitro acyltransferase assays) is not yet available due to current technical constraints.

C4 is a multifunctional viral protein that has evolved sophisticated mechanisms to suppress plant immune responses. Previous studies have shown that C4 disrupts host brassinosteroid (BR) signaling through membrane-associated interactions to facilitate viral infection. For example, the C4 protein of beet curly top virus (BCTV) undergoes N-myristoylation, which anchors it to the plasma membrane. There, it interacts with several *Arabidopsis* Shaggy-like kinases (AtSKs), promoting its phosphorylation and activation, ultimately compromising host cell cycle regulation (Mills-Lujan et al. 2015). Similarly, the C4 protein of sweet potato leaf curl virus-Jiangsu (SPLCV-JS) binds to brassinosteroid-insensitive 2 (AtBIN2) at the plasma membrane. This interaction triggers the nuclear relocalization of AtBIN2-interacting transcription factors AtBES1 and AtBZR1, altering the expression of BR-responsive genes and activating BR signaling (Bi et al. 2017). Beyond targeting the BR pathway, C4 proteins from TYLCV, BCTV, and East African cassava mosaic virus (EACMV) relocalize from the plasma membrane to chloroplasts upon artificial induction of pattern-triggered immunity. This relocalization interferes with salicylic acid (SA)-mediated defense responses (Medina-Puche et al. 2020). Additionally, the C4 protein from cotton leaf curl Multan virus (CLCuMuV) binds to the eukaryotic translation initiation factor eIF4A, a negative regulator of autophagy. By enhancing the interaction between eIF4A and autophagy-related protein 5 (ATG5), C4 inhibits autophagy pathways and facilitates viral infection (Yang et al. 2023). In our study, we found that the C4 of TYLCCxV specifically interacts with the palmitoyl acyltransferase NbPAT4. However, the molecular mechanism by which C4 exploits the host S-acylation machinery—and whether S-acylated C4 engages additional host factors (e.g., BAM1, SnRK1, eIF4A, SKη kinase) to suppress multiple layers of plant immunity—remains unclear and warrants further investigation.

To date, the enzymatic regulation of depalmitoylation in plant-pathogen interactions remains largely uncharacterized, and functional homologs of the ABHD17 family have not been identified in plant species beyond *A. thaliana*. Recently, ABAPT3 in *Arabidopsis* was reported to mediate the depalmitoylation of the C4 protein from BSCTV (Zhao et al. 2024). In our study, we identified the de-S-acylation enzyme NbABHD6 in *N. benthamiana* as a C4 interactor of TYLCCxV (Fig. 5A–D). We further demonstrated that NbABHD6-mediated depalmitoylation reduced the S-acylation level of C4 (Fig. 6 A, B). In summary, our findings reveal that the C4 protein undergoes dynamic S-acylation regulation mediated by two host enzymes. Palmitoyl acyltransferase NbPAT4 facilitates C4 membrane anchoring via S-palmitoylation, while NbABHD6 catalyzes its depalmitoylation, highlighting the reversible nature of this post-translational modification.

Previous studies have shown that S-acylation influences protein stability and accumulation in various systems, including SNARE (soluble N-ethylmaleimide-sensitive factor attachment protein receptor) complexes and anthrax toxin receptors (Linder and Deschenes 2007). More recent findings demonstrate that S-palmitoylation of the plant immune receptor kinase FLS2 stabilizes its interaction with the co-receptor BAK1 (BRI1-associated receptor kinase 1) at the plasma membrane, thereby preventing FLS2 from entering the endocytic pathway (Hurst et al. 2023). In our study, we observed that co-infiltration of NbABHD6 reduced C4 protein accumulation (Fig. 7A, B), suggesting that NbABHD6 negatively regulates C4 levels. However, whether this reduction depends on the depalmitoylase activity of NbABHD6 remained unclear. It is known that members of the ABHD17 family rely on a catalytic triad—comprising serine (S), aspartate (D), and histidine (H)—for depalmitoylase activity (Yokoi et al. 2016). In *Arabidopsis*, ABAPT8 contains three conserved catalytic residues (S147, D212, and H241), and the catalytic mutant ABAPT8 (mSHD), in which these residues are substituted with alanine, loses the ability to affect the S-acylation of the immune-related protein RIN4 (Liu et al. 2021). Consistent with this mechanism, our bioinformatic analysis identified the same catalytic triad—S147, D212, and H241—in NbABHD6 (Fig. 5E), indicating a conserved enzymatic function among ABHD6 homologs. We further demonstrated that NbABHD6 regulates C4 accumulation through its depalmitoylase activity. Both the S-acylation level and protein accumulation of C4 remained unchanged when co-expressed with either GFP or the catalytically inactive NbABHD6 mutant (mSDH)-GFP (Fig. 7C, D). In vivo degradation inhibition assays revealed that C4 undergoes proteasomal degradation via the 26S proteasome (Fig. 7F). Moreover, NbABHD6-mediated depalmitoylation promotes C4 turnover through the ubiquitin–proteasome system (Fig. 7G, H), thereby modulating its protein abundance.

Researchers typically assess protein S-acylation by evaluating both S-acylation levels and subcellular localization (Liu et al. 2021). In our study, we found that NbABHD6 did not significantly alter the plasma membrane localization of C4 (data not shown), possibly due to the presence of a myristoylation site at the second amino acid position of C4 (Supplemental Fig. S1A). Additionally, the S-acylation-deficient mutant C4^C4S^ (Fig. 1E) remained partially plasma membrane localization (Fig. 1A–B), indicating that C4 membrane association is also mediated by other modifications, such as myristoylation. These observations suggest that subcellular localization alone does not reliably reflect the S-acylation status of C4. Therefore, we employed the biotin switch assay to directly assess S-acylation levels of C4 regulated by NbABHD6. To comprehensively validate the S-acylation state of C4, we combined several complementary approaches, including bioinformatic prediction, subcellular localization analysis, the biotin switch assay, and mass spectrometry.

S-palmitoylation may act synergistically with other post-translational modifications, including phosphorylation, ubiquitination, and S-nitrosylation. For example, S-acylation of PD-L1 (programmed death ligand 1) at cysteine 272 suppresses its ubiquitination and prevents cytoplasmic trafficking to multivesicular bodies (Yang et al. 2019; Yao et al. 2019). In *Arabidopsis*, the immune receptor P2K1 phosphorylates the palmitoyl acyltransferases PAT5 and PAT9, enhancing their activity of S-acyltransferase toward P2K1 itself and attenuating immune signaling (Chen et al. 2021). Certain modifications, such as S-nitrosylation and oxidation, compete for cysteine residues with S-palmitoylation (Lennicke and Cochemé 2021). The interplay between S-nitrosylation and S-acylation influences the trafficking and localization of post-synaptic density proteins, destabilizing synaptic networks (Zareba-Koziol et al. 2019). In addition, S-acylation plays a central role in plant defense pathways. In *Arabidopsis*, S-acylation of R5L1 (resistance to *Pseudomonas syringae* 5-like 1) by PAT13 and PAT16 is essential for R5L1 membrane localization and activation of immune responses (Gao et al. 2022). SA modulates the localization of brassinosteroid signaling kinases (BSKs) by reducing their S-acylation levels through ABAPT11-mediated de-S-acylation. This mechanism integrates SA and BR signaling to coordinate plant development (Liu et al. 2023). Our findings demonstrate that NbABHD6-mediated depalmitoylation facilitates C4 degradation via the ubiquitin–proteasome system (UPS). Nevertheless, key mechanistic questions remain unresolved: (1) Does C4 recruit specific E3 ubiquitin ligase complexes to initiate UPS-mediated degradation? (2) How does S-acylation coordinate with ubiquitination or myristoylation to form a lipid modification network that facilitates viral suppression of plant immunity? Future studies employing omics approaches will be instrumental in systematically identifying co-modified proteins or uncovering crosstalk between S-acylation and other post-translational modifications on C4.

The current understanding of dynamic S-acylation regulation is primarily based on animal systems. In mammals, Signal Transducer and Activator of Transcription 3 (STAT3) undergoes a series of post-translational modifications to regulate immune homeostasis: (1) DHHC7-mediated S-palmitoylation at Cys108 anchors STAT3 to the plasma membrane; (2) Phosphorylation by Janus kinase 2 (JAK2) at Tyr705 primes its activation; (3) APT2-catalyzed depalmitoylation drives nuclear translocation, disrupting TH17 cell differentiation and contributing to chronic inflammatory bowel disease (Zhang et al. 2020; Wei et al. 2024). In contrast, the regulation of plant S-acylation cycling—especially in the context of antiviral immunity—remains largely unexplored.

**Fig. 8:**
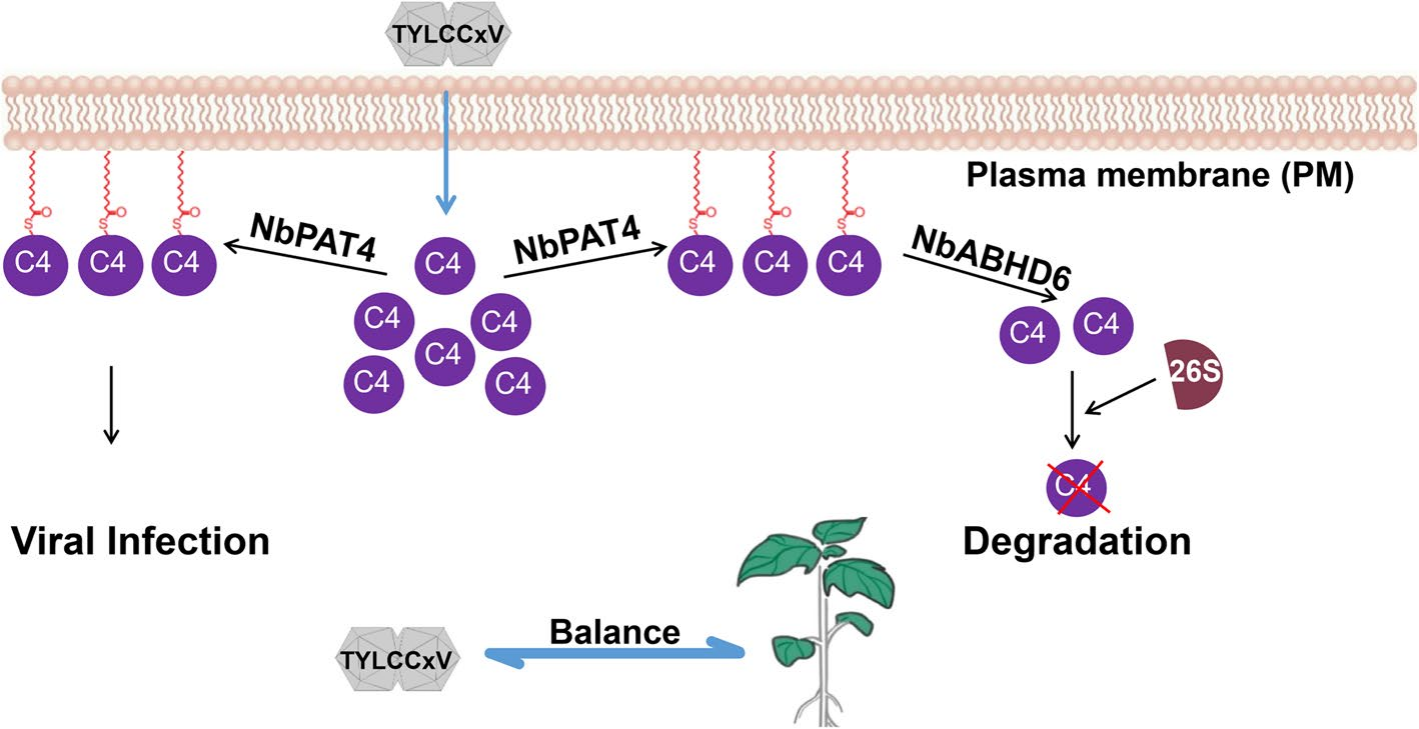
A model of C4 S-palmitoylation by the palmitoyl acyltransferase NbPAT4 and the depalmitoylase NbABHD6 orchestrates geminiviral infection dynamics

This study presents the first characterization of the S-acylation cycle during plant-geminivirus interactions, identifying the host-encoded palmitoyl acyltransferase NbPAT4 and depalmitoylase NbABHD6 as key regulators of TYLCCxV C4 modification. Based on these findings, we propose a mechanistic model in which the S-acylation cycle governs viral pathogenesis. During early TYLCCxV infection, the viral effector C4 exploits the host S-acylation machinery by interacting with NbPAT4. This modification anchors C4 at the plasma membrane and enhances its stability, thereby promoting viral accumulation and infection. In response, the host plant deploys the depalmitoylase NbABHD6 to counteract this strategy. NbABHD6-mediated depalmitoylation reduces C4 S-acylation levels, triggering its degradation via the ubiquitin–proteasome pathway and suppressing viral infection (Fig. 8). Our findings align with the recently proposed concept of apoplastic interactive balance (AIB) (Liu et al. 2026), which conceptualizes the plant–pathogen interface as a dynamic system maintained by reciprocal molecular adjustments. Within this framework, the reciprocal regulation of C4 by NbPAT4 and NbABHD6 represents a host–pathogen enzymatic tug-of-war at the post-translational level. The virus tilts the balance in its favor by exploiting host S-acylation machinery (NbPAT4) to stabilize C4, whereas the host restores equilibrium by deploying a counteracting depalmitoylase (NbABHD6), which targets C4 for proteasomal degradation. This ongoing cycle of imbalance and restoration—a core tenet of the AIB model—not only governs geminiviral pathogenicity but also illustrates how such local enzymatic conflicts maintain system-level functional stability in the apoplast.

## Conclusion

In this study, we demonstrate that the TYLCCxV C4 protein undergoes reversible S-acylation at cysteine residue 4. The palmitoyl acyltransferase NbPAT4 catalyzes C4 palmitoylation, promoting its accumulation and plasma membrane localization, thereby enhancing viral infection. Conversely, the depalmitoylase NbABHD6 mediates C4 de-S-acylation, leading to its degradation via the 26S proteasome pathway. This dynamic palmitoylation–depalmitoylation cycle of C4 reveals a previously unrecognized regulatory layer in the arms race between geminiviruses and host defenses. Based on these findings, we propose a new model of plant-geminivirus interaction centered on the S-palmitoylation cycle. This cycle exemplifies the concept of AIB and supports a host–pathogen enzymatic tug-of-war model, wherein the virus (via NbPAT4) and the host (via NbABHD6) compete for control of C4 stability. Such enzymatic conflict drives the dynamic equilibrium that underlies geminivirus–plant coevolution.

## Materials and methods

### Plant material and growth condition

Transgenic *Nicotiana benthamiana* plants expressing the nuclear marker H2B-RFP (a full-length red fluorescent protein fused to the C-terminus of histone 2B) were kindly provided by Dr. Michael M. Goodin (University of Kentucky, KY, USA) (Martin et al. 2009). The transgenic *NbPAT4* knockout (*Nbpat4*) and overexpressing (OE-NbPAT4) *N. benthamiana* plants were generated by transforming with the binary vectors BGK01-NbPAT4 and pCAMBIA-NbPAT4 (Biorun, Wuhan, China). Single guide RNA targeting the NbPAT4 open reading frame was designed using CRISPR-P tool V2.0 (http://crispr.hzau.edu.cn/CRISPR2/) and listed in Supplemental Table S2. T2 homozygous transgenic plants were confirmed positive before virus inoculation. Plants were grown in an insect-free growth incubator at 25 °C with a 16-h light/8-h dark photoperiod.

### Construction of plasmids

NbPAT4 was cloned into pPR3-N, p2YC, and pCambia-2 × 35S-3 × Flag vectors, respectively. NbABHD6 was individually cloned into pPR3-N, p2YC, and pCambia-2 × 35S-GFP vectors, and the NbABHD6 (mSDH) mutant was cloned into pPR3-N and pCambia-2 × 35S-GFP vectors. NbPAT1-2, NbPAT6, NbAPT1-4, and NbABHD1-15 were cloned into pPR3-N vectors. TYLCCxV C4 was cloned into pDHB1, p2YN, pCambia-2 × 35S-GFP, pCambia-2 × 35S-3 × Flag, and PVX vector pGR106, respectively. The C4 (C4S) mutant was cloned into pGR106, pCambia-2 × 35S-GFP, and pCambia-2 × 35S-3 × Flag. All fragments were PCR-amplified using KOD One™ PCR Master Mix (Toyobo, Shanghai, China). The resulting PCR products were inserted into vectors via homologous recombination using the MonClone™ Single Assembly Cloning Mix (Monad, Suzhou, China) and verified by DNA sequencing. The NbABHD6 (mSDH) mutant was generated using overlapping PCR, as described by Tao et al. (2002). All primers used for plasmid construction are listed in Supplemental Table S2.

### Construction of infectious clones

The infectious clone of TYLCCxV (pBinPLUS-TYLCCxV-1.4A) and the plasmid pLB-TYLCCxV-1A were previously described (Xie et al. 2024). The mutant pLB-TYLCCxV (C4^C4S^)−1A was generated by introducing the C4S mutation in C4 using homologous recombination (Monad, Suzhou, China). After sequencing, the *Kpn*I-*Cla*I fragment from pLB-TYLCCxV (C4^C4S^)−1A, including the intergenic region, was cloned into the binary vector pBinPLUS to produce pBinPLUS-TYLCCxV (C4^C4S^)−0.4A. The full-length *Kpn*I-digested fragment of pLB-TYLCCxV (C4^C4S^)−1A was inserted into the unique *Kpn*I site of pBinPLUS-TYLCCxV (C4^C4S^)−0.4A to generate pBinPLUS-TYLCCxV (C4^C4S^)−1.4A. This construct was then transformed into *Agrobacterium tumefaciens* strain EH105 to create the infectious clone of the mutant virus, TYLCCxV (C4^C4S^).

### Viral inoculation and agroinfiltration

For geminivirus agroinoculation, *Agrobacterium* cultures carrying the infectious clones of TYLCCxV or TYLCCxV (C4^C4S^) were infiltrated into *N. benthamiana* plants at the 5- to 6-leaf stage. Plants infiltrated with the empty pBinPLUS vector served as mock controls. For the PVX assay, the recombinant PVX::C4 and PVX::C4^C4S^ constructs were introduced into *A. tumefaciens* strain GV3101 via electroporation. *A. tumefaciens* carrying the empty PVX vector pGR106 (PVX) was used as a negative control. Prior to infiltration, the optical density (OD_600_) of each Agrobacterium culture was adjusted to 0.8–1.0. Each treatment was infiltrated into 12 plant seedlings, and the experiments were performed in triplicate biological replicates.

### Yeast Split-ubiquitin assay

Yeast transformations were performed in NMY51 cells according to a DUALhunter Starter Kit (Takara, Dalian, China). Transformants were grown on synthetic medium (SD-Trp/-Leu) at 30 °C for 72 h. Subsequently, the cultures were transferred to selective medium (SD-Trp/-Leu/-His/-Ade) to assess binding activity, and the experiments were performed in triplicate biological replicates.

### BiFC Assay

The BiFC assay was conducted as described by Li et al. (2020). 2YN-C4 and 2YC-NbPAT4 or 2YC-NbABHD6 were co-infiltrated into transgenic *H2B-RFP* N. benthamiana leaves at the 5- to 6-leaf stage. The plants were examined at 48 hpi in the epidermal cells of 1–2 cm^2^ leaf explants using a Zeiss LSM780 laser confocal microscope (Zeiss, Oberkochen, Germany). The experiments were performed in triplicate biological replicates.

### Co-IP assay

*N. benthamiana* leaves co-infiltrated with C4-GFP and NbPAT4-Flag or C4-Flag and NbABHD6-GFP were harvested at 48 hpi. The leaves were ground in 1 mL IP buffer (50 mM Tris–HCl, 150 mM NaCl, 10 mM MgCl₂, 5 mM DTT, and 0.1% Triton X-100), then centrifuged at 8,000 g for 15 min at 4 °C. Immunoprecipitation was performed using anti-GFP mAb magnetic beads (MBL, Beijing, China), as previously described (Mei et al. 2018). The experiments were performed in triplicate biological replicates.

### Subcelluar localization and fractionation

Transgenic *H2B-RFP N. benthamiana* plants were infiltrated with C4-GFP, C4^C4S^-GFP, or GFP. Fluorescence signals were captured at 48 hpi using a Zeiss LSM780 laser confocal microscope (Zeiss, Oberkochen, Germany). At 48 hpi, infiltrated *N. benthamiana l*eaves were ground in homogenization buffer (50 mM Tris–HCl, pH 8.0, 10 mM KCl, 3 mM MgCl₂, 1 mM EDTA, 1 mM DTT, 0.1% BSA, 0.3% dextran, and 13% [w/v] sucrose). The protein samples were centrifuged at 3,000 g for 20 min at 4 °C to remove nuclei and large cellular debris. The supernatant was then ultracentrifuged at 30,000 g for 1 h at 4 °C to separate the soluble (S30) and pellet (P30) fractions. All fractions were analyzed by SDS-PAGE and Western blotting as previously described (Mei et al. 2018). GFP and PIP2A-DsRed (plasma membrane intrinsic protein 2A, with intact DsRed fused to its C-terminus) served as markers for the soluble and membrane-enriched fractions, respectively. The experiments were performed in triplicate biological replicates.

### LC–MS/MS and biotin-switch assay

*N. benthamiana* leaves were infiltrated with C4-Flag, and the protein extracts were analyzed by SDS-PAGE followed by Q-Exactive LC–MS/MS using a Q-Exactive HF system (Jingjie, Hangzhou, China). S-acylation assays of C4 protein were conducted as described by Hemsley et al. (2008) and Yue et al. (2022). Protein extracts were treated with hydroxylamine (Hyd) to selectively cleave the sulfur bond, then combined with biotin-HPDP (Thermo Scientific, Shanghai, China). The proteins were incubated with neutravidin-agarose beads (Thermo Scientific), and the final protein samples were analyzed by Western blotting using anti-Flag and anti-GFP antibodies, and the experiments were performed in triplicate biological replicates.

### RNA extraction, Quantitative RT-PCR (qRT-PCR), and Quantitative real-time PCR (qPCR)

Total RNA was extracted from *N. benthamiana* leaves using TRIzol reagent (Takara, Dalian, China), following the manufacturer’s instructions. cDNA was synthesized with the ReverTra Ace qPCR RT Master Mix with gDNA Remover (Toyobo, Osaka, Japan). qPCR was performed using ChamQ SYBR Color qPCR Master Mix (Vazyme, Nanjing, China), and expression levels were normalized to Nbactin. The experiments were performed in triplicate biological replicates, and the specific primers used in the experiment are listed in Supplemental Table S2.

### DNA extraction and Southern blot analysis

Total DNA was extracted using the cetyl trimethyl ammonium bromide (CTAB) method, and Southern blot analysis was performed as described by Li et al. (2024). After denaturation and neutralization, the DNA samples were transferred to Hybond N + nylon membranes (GE Healthcare, Pittsburgh, PA, USA). Viral genomic DNA was detected by hybridization with a TYLCCxV coat protein digoxin-labeled probe, using a DIG High Prime DNA Labeling and Detection Starter Kit II (Roche Diagnostics, Mannheim, Germany).

### Protein extraction and western blotting

Total protein extraction and Western blotting were carried out as described by Porcellato et al. (2022). Immunoblotting was performed using Mouse anti-GFP-Tag monoclonal antibody (ABclonal, Wuhan, China), Mouse anti-Flag-Tag monoclonal antibody (ABclonal), and HRP-conjugated Goat anti-Mouse or anti-Rabbit IgG antibody (ImmunoWay, Suzhou, China). The experiments were performed in triplicate biological replicates.

### Chemical treatments

*N. benthamiana* leaves were infiltrated with C4-GFP and treated with 500 μM 2-BP (a palmitoylation inhibitor) (Aladdin, Shanghai, China) or CH_3_OH to assess the effect of 2-BP on subcellular localization. For the protein degradation assay, 100 μM MG132 (a 26S proteasome pathway inhibitor) (MCE, Shanghai, China), 100 μM E64d (MCE), 5 mM 3-MA (autophagy pathway inhibitor) (MCE), or DMSO combined with 100 μM CHX (MCE) were co-infiltrated with C4-Flag into *N. benthamiana* leaves. After 4 h, samples were harvested as previously described (Li et al. 2024). This experiments were performed in triplicate biological replicates.

## Supplementary Information

Supplementary Material 1: Fig. S1. Structure of C4 protein as predicted by an online database. Fig. S2. Alignment of ABHDs from different ABHD17 family members as analyzed by Mega 7.0. Table S1. De-S-acylation enzymes applying for Yeast Split-ubiquitin assay. Table S2. Primers and their sequence used in this study.

## Data Availability

All data and materials are available in the paper and online supplemental files.
