# Supplementary material for: Reversible S-palmitoylation of C4 protein encoded by TYLCCxV orchestrates geminiviral pathogenesis
Source: Stress Biol. 2026 May 6;6(1):35. doi: 10.1007/s44154-026-00308-2 (PMC13144479; doi:10.1007/s44154-026-00308-2)
Supplement: Supplementary file 1 — Supplementary Material 1: Fig. S1. Structure of C4 protein as predicted by an online database. Fig. S2. Alignment of ABHDs from different ABHD17 family members as analyzed by Mega 7.0. Table S1. De-S-acylation enzymes applying for Yeast Split-ubiquitin assay. Table S2. Primers and their sequence used in this study. [file 44154_2026_308_MOESM1_ESM.docx]

**
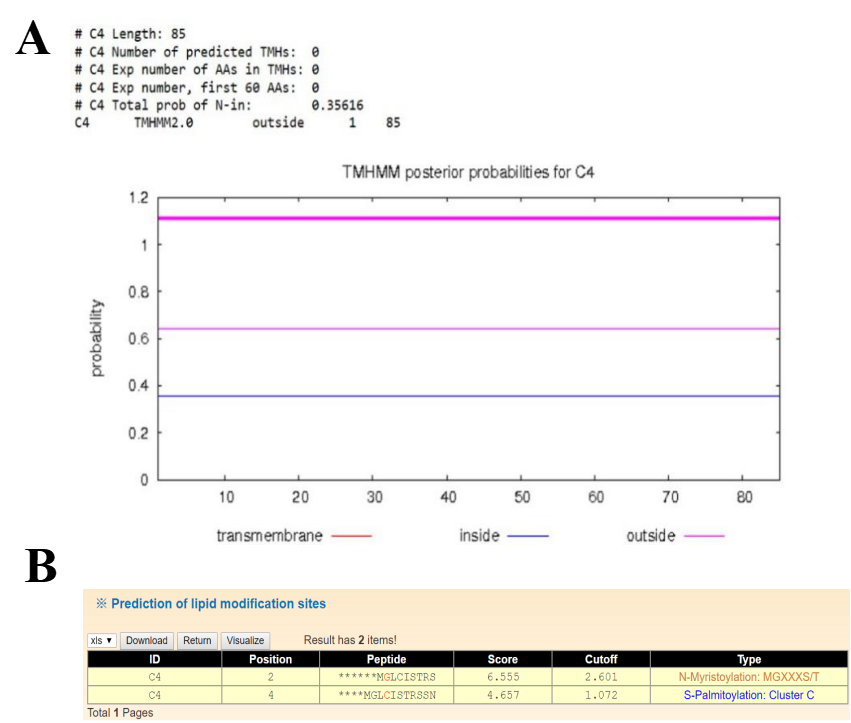
**

**Fig. S1** Structure of C4 protein as predicted by an online database. **A** Transmembrane domain of C4 protein as predicted by an online database (<http://www.cbs.dtu.dk/services/TMHMM>). **B** Lipidation modification of C4 protein as predicted by the CSS-Palm 4.0 online server (<http://csspalm.biocuckoo.org/online>.php). N-Myristoylation at glycine-2 and S-Palmitoylation at cysteine-4 in C4 were predicted.

**
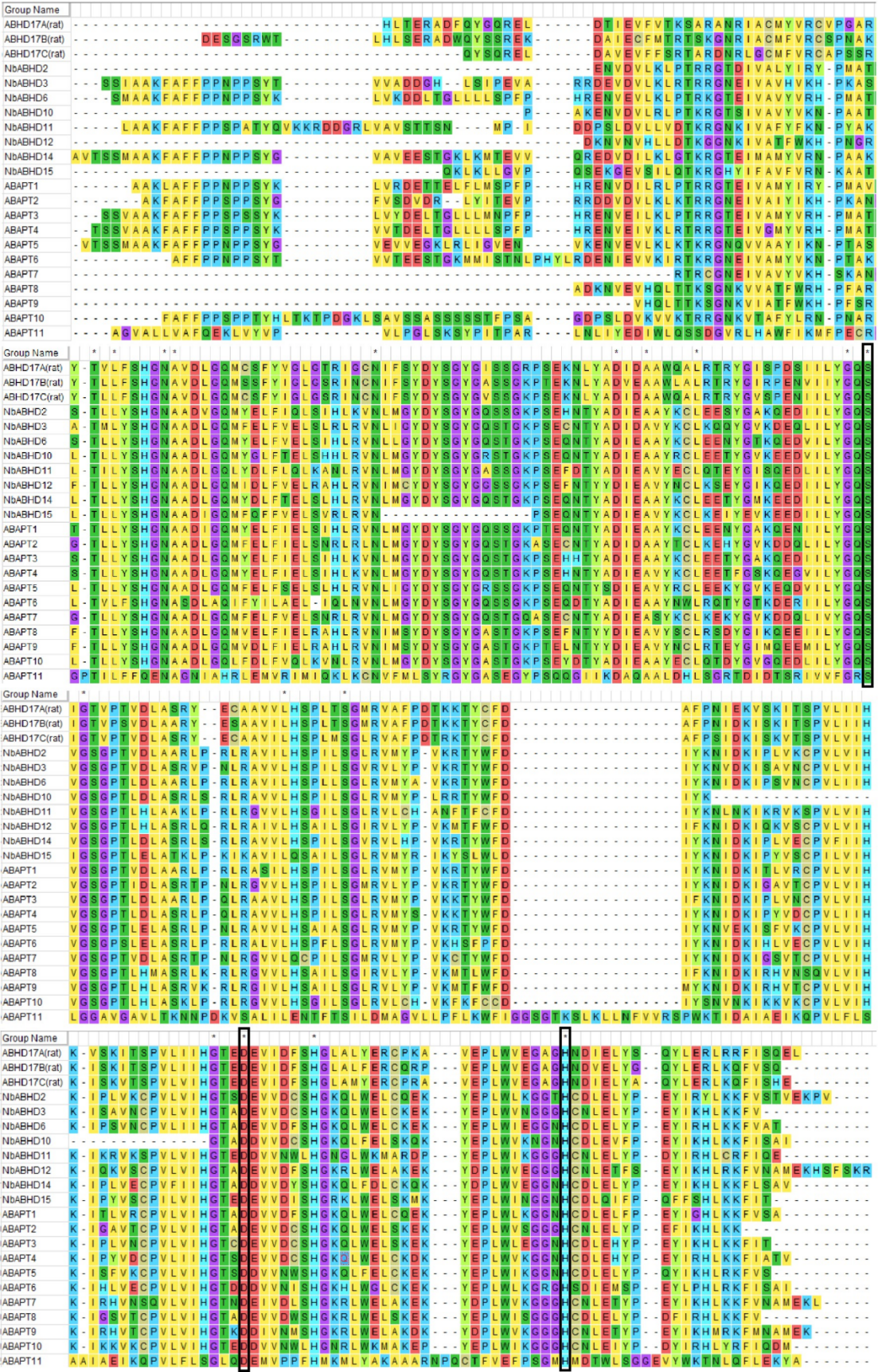
**

**Fig.** **S2** Alignment of ABHDs from different ABHD17 family members as analyzed by Mega 7.0. The ABHD regions of rat ABHD17A/B/C, NbABHD2-3, NbABHD6, NbABHD10-12, and NbABHD14-15 from *N. benthamiana* and ABAPT1-11 from *Arabidopsis* were analyzed for amino acid sequence alignment using ClustalW. Three conserved residues, serine (S), aspartate (D), and histidine (H), are indicated by the black box.

**Table S1 De-S-acylation enzymes applying for Yeast Split-ubiquitin assay**

| Depalmitoylase | Accession Number |
| --- | --- |
| NbAPT1 | Niben101Scf03209g00028.1 |
| NbAPT2 | Niben101Scf00349g02005.1 |
| NbAPT3 | Niben101Scf15979g02003.1 |
| NbAPT4 | Niben101Scf00123g06029.1 |
| NbABHD1 | Niben101Scf02287g00002.1 |
| NbABHD2 | Niben101Scf02159g01008.1 |
| NbABHD3 | Niben101Scf02381g08018.1 |
| NbABHD4 | Niben101Scf00736g00015.1 |
| NbABHD5 | Niben101Scf00507g00008.1 |
| NbABHD6 | Niben101Scf03766g07007.1 |
| NbABHD7 | Niben101Scf02358g01009.1 |
| NbABHD8 | Niben101Scf04138g01017.1 |
| NbABHD9 | Niben101Scf12270g00013.1 |
| NbABHD10 | Niben101Scf02738g07005.1 |
| NbABHD11 | Niben101Scf01800g02008.1 |
| NbABHD12 | Niben101Scf01789g04012.1 |
| NbABHD13 | Niben101Scf02073g05007.1 |
| NbABHD14 | Niben101Scf09115g00016.1 |
| NbABHD15 | Niben101Scf04187g01012.1 |

**Table S2 Primers and their sequence used in this study**

| Primer | Sequence |
| --- | --- |
| sgRNA | GTCTCCCGAGATAAGAACTA |
| 2YN-C4-R | ACCTCCTCCACTAGTGGGCCTCTGCTGCTGCA |
| C4-GFP-F | ACGGGGGACGAGCTCGGTACCATGGGTCTCTGCATATCCACGC |
| C4(C4S)-GFP-F | ACGGGGGACGAGCTCGGTACCATGGGTCTCTCCATATCCACGC |
| C4-GFP/C4(C4S)-GFP-R | GCCCTTGCTCACCATGTCGACGGGCCTCTGCTGCTGCA |
| C4-Flag/C4(V14A)-Flag-F | GAGCTCGGTACCCGGGGATCCATGGGTCTCTGCATATCCACGCGC |
| C4(C4S)-Flag-F | GAGCTCGGTACCCGGGGATCCATGGGTCTCTCCATATCCACGC |
| C4-/C4S-/ V14A-Flag-R | GTCGTCGACTCTAGAGGATCCGGGCCTCTGCTGCTGCA |
| pPR3-N-NbPAT4-F | GTTCCAGATTACGCTGGATTCATGTATGTGGTACCTCCTCCG |
| pPR3-N-NbPAT4-R | GATAAGCTTGATATCGAATTCTCACAAGTTTTGATCAGTTGGTTGG |
| pPR3-N-NbPAT6-F | GTTCCAGATTACGCTGGATTCATGTATGGGGTGGCTCCG |
| pPR3-N-NbPAT6-R | GATAAGCTTGATATCGAATTCCTATTGCCCGTTTTCAGTTGGG |
| 2YC-NbPAT4-F | TTACGAACGATAGTTAATTAAATGTATGTGGTACCTCCTCCG |
| 2YC-NbPAT4-R | GTGATTTTTGCGGACTCTAGATCACAAGTTTTGATCAGTTGGTTGG |
| NbPAT4-Flag-F | GAGCTCGGTACCCGGGGATCCATGTATGTGGTACCTCCTCCG |
| NbPAT4-Flag-R | GTCGTCGACTCTAGAGGATCCTCACAAGTTTTGATCAGTTGGTTGG |
| pDHB1-C4-F | ATTACGGCCAGGCCTCCATGGATGGGTCTCTGCATATCCACGC |
| pDHB1-C4-R | GGCGGCCAAGATATAGGGCCTCTGCTGCTGCA |
| pPR3-N-NbAPT1-F | GTTCCAGATTACGCTGGATTCATGAAATTCATTTTTCCATTACTAAAACC |
| pPR3-N-NbAPT1-R | GATAAGCTTGATATCGAATTCAGAATTGCTTTGAAGACGAGTTTTG |
| pPR3-N-NbAPT2-F | GTTCCAGATTACGCTGGATTCATGAGCTTCAGTGGCTCTACAGC |
| pPR3-N-NbAPT2-R | GATAAGCTTGATATCGAATTCTGTCCCCCCAAGACTCAAATT |
| pPR3-N-NbAPT3-F | GTTCCAGATTACGCTGGATTCATGAGTTATATGAATCCTTCCATGAGC |
| pPR3-N-NbAPT3-R | GATAAGCTTGATATCGAATTCTCGTCGTAAACCTTCAAGTCCA |
| pPR3-N-NbAPT4-F | GTTCCAGATTACGCTGGATTCATGAGTTATTTAAACCCTTCAACTGG |
| pPR3-N-NbAPT4-R | GATAAGCTTGATATCGAATTCCATTCCATGAGAGAGTAGAATAGGTAAAG |
| pPR3-N-NbABHD1-F | GTTCCAGATTACGCTGGATTCATGGCCGCGTTTAAGGAAA |
| pPR3-N-NbABHD1-R | GATAAGCTTGATATCGAATTCGCTTGTTGAATTACTCCATATTTCCT |
| pPR3-N-NbABHD2-F | GTTCCAGATTACGCTGGATTCATGGGTGGGGTAACGTCGTC |
| pPR3-N-NbABHD2-R | GATAAGCTTGATATCGAATTCACCAGCTCTTATTCTATCTAGCCAGTC |
| pPR3-N-NbABHD3-F | GTTCCAGATTACGCTGGATTCATGGGGGGAGTGACTTCTTCTAT |
| pPR3-N-NbABHD3-R | GATAAGCTTGATATCGAATTCCCACACTAATCCCTTTCTTCTCCT |
| pPR3-N-NbABHD4-F | GTTCCAGATTACGCTGGATTCATGGTGTCGTACGTGAGCTTGTT |
| pPR3-N-NbABHD4-R | GATAAGCTTGATATCGAATTCGCTCGCGACAAATTCTGAAGAT |
| pPR3-N-NbABHD5-F | GTTCCAGATTACGCTGGATTCATGGAACAACTTGTCAACTTTATTATTAGA |
| pPR3-N-NbABHD5-R | GATAAGCTTGATATCGAATTCTCTGTTCTTGAAAAAGTTGAGATCCC |
| pPR3-NbABHD6/mSDH-F | GTTCCAGATTACGCTGGATTCATGGGAGGGGTAACCTCATCA |
| pPR3-NbABHD6/mSDH-R | GATAAGCTTGATATCGAATTCCCCCGTTCGTATTCTCTCCAT |
| pPR3-N-NbABHD7-F | GTTCCAGATTACGCTGGATTCATGGGGGAGGTCAATGGGT |
| pPR3-N-NbABHD7-R | GATAAGCTTGATATCGAATTCGGAGGAGCTGTATCTCCTATGGTT |
| pPR3-N-NbABHD8-F | GTTCCAGATTACGCTGGATTCATGGATACTTGTCTACTTCATATGCAAC |
| pPR3-N-NbABHD8-R | GATAAGCTTGATATCGAATTCGAACTTTGTATAGCCTTGCAAGACA |
| pPR3-N-NbABHD9-F | GTTCCAGATTACGCTGGATTCATGTCGAGCATCTGCAGAACC |
| pPR3-N-NbABHD9-R | GATAAGCTTGATATCGAATTCAACAAGAGAAGCTAATTTGGGAGTTA |
| pPR3-N-NbABHD10-F | GTTCCAGATTACGCTGGATTCATGGGGGGAGTAACGTCATCG |
| pPR3-N-NbABHD10-R | GATAAGCTTGATATCGAATTCGCAAGGGTAATCTAACCGGTCA |
| pPR3-N-NbABHD11-F | GTTCCAGATTACGCTGGATTCATGGGTTGTTGTTTATCTCAGTTAGC |
| pPR3-N-NbABHD11-R | GATAAGCTTGATATCGAATTCCCAACACGAACATTTTACACTCCA |
| pPR3-N-NbABHD12-F | GTTCCAGATTACGCTGGATTCATGGGGAATGTAACAGCAAGTGT |
| pPR3-N-NbABHD12-R | GATAAGCTTGATATCGAATTCTCTCTTTCCAAATCTTAAGCATCTGT |
| pPR3-N-NbABHD13-F | GTTCCAGATTACGCTGGATTCATGTCGGCCATTTCGAATTTC |
| pPR3-N-NbABHD13-R | GATAAGCTTGATATCGAATTCAAATCCTTGGAAGGAAAATGAAAG |
| pPR3-N-NbABHD14-F | GTTCCAGATTACGCTGGATTCATGGGAGCTGTGACGTCGTC |
| pPR3-N-NbABHD14-R | GATAAGCTTGATATCGAATTCTTTCGATGTTTCAAGTTTATCTATTTGG |
| pPR3-N-NbABHD15-F | GTTCCAGATTACGCTGGATTCATGGGTGGTGCAGCATCAAC |
| pPR3-N-NbABHD15-R | GATAAGCTTGATATCGAATTCGTCCTCCTCAGATAGTGTCTGCTTC |
| 2YC-NbABHD6-F | TTACGAACGATAGTTAATTAAATGGGAGGGGTAACCTCATC |
| 2YC-NbABHD6-R | GTGATTTTTGCGGACTCTAGAGCTTTTCCTAGAACTGATCCTCTG |
| NbABHD6/ mSDH-GFP-F | ACGGGGGACGAGCTCGGTACCATGGGAGGGGTAACCTCATC |
| NbABHD6 / mSDH-GFP-R | GCCCTTGCTCACCATGTCGACGCTTTTCCTAGAACTGATCCTCTGT |
| NbABHD6 (mSDH)-F1 | CAAGCAGTTGGTAGTGGGC |
| NbABHD6 (mSDH)-R1 | ACTACCAACTGCTTGGCC |
| PVX-C4-F | GCTAGCATCGATTGGCGCGCCATGGGTCTCTGCATATCCACGC |
| PVX-C4(C4S)-F | GCTAGCATCGATTGGCGCGCCATGGGTCTCTCCATATCCACGC |
| PVX-C4/PVX-C4(C4S)-R | AACCGTTCATCGGCGGTCGACGGGCCTCTGCTGCTGCA |
| pLB-TYLCCxV(C4S)1A-F | ATGGGTCTCTCCATATCCACGC |
| pLB-TYLCCxV(C4S)1A-R | GTGGATATGGAGAGACCCATCTTGGTG |
| pBinPLUS-TYLCCxV1.4AF | ATTAAAATTGGCGGCGGTACCCCTAATGATTTTCAGCAGGTTTTTAATG |
| pBinPLUS-TYLCCxV1.4AR | CTATGCACATATATTAATTAAGGTAGGACGCCTGTCCCGG |
| probe-F | AATAAAGGGGATTTTGAATCTCCCAG |
| probe-R | CCAATTCAAAGGTAAATGCCAG |
| qPCR-TRV-NbABHD6-F | CCTTAAGTTGCCCACTCGCA |
| qPCR-TRV-NbABHD6-R | CGCGCAAGTGAATGCTCAAT |
| qPCR-TYLCCxV-F | CTTCTTGGCTAGCCTGTGCT |
| qPCR-TYLCCxV-R | TGCAATCCAGGACCCAACTC |
